# Genome analysis of *Cephalotrichum gorgonifer* and identification of the biosynthetic pathway for rasfonin, an inhibitor of KRAS dependent cancer

**DOI:** 10.1186/s40694-023-00158-x

**Published:** 2023-06-24

**Authors:** Andreas Schüller, Lena Studt-Reinhold, Harald Berger, Lucia Silvestrini, Roman Labuda, Ulrich Güldener, Markus Gorfer, Markus Bacher, Maria Doppler, Erika Gasparotto, Arianna Gattesco, Michael Sulyok, Joseph Strauss

**Affiliations:** 1 Department of Applied Genetics and Cell Biology, Institute of Microbial Genetics, University of Natural Resources and Life Sciences, Vienna (BOKU), Campus Tulln, Konrad Lorenz Strasse 24, 3430 Tulln an der Donau, Austria; 2 Research Platform Bioactive Microbial Metabolites (BiMM), Konrad Lorenz Strasse 24, 3430 Tulln an der Donau, Austria; 3 Department for Farm Animals and Veterinary Public Health, Institute of Food Safety, Food Technology and Veterinary Public Health, Unit of Food Microbiology, University of Veterinary Medicine Vienna, Veterinaerplatz 1, 1210 Vienna, Austria; 4 Department of Bioinformatics, Technical University of Munich, TUM School of Life Sciences Weihenstephan, Freising, Germany; 5 AIT Austrian Institute of Technology GmbH, Bioresources, 3430 Tulln, Austria; 6 Department of Chemistry, Institute of Chemistry of Renewable Resources, University of Natural Resources and Life Sciences Vienna (BOKU), Konrad-LorenzStraße 24, 3430 Tulln, Austria; 7 Department of Agrobiotechnology (IFA-Tulln), Institute of Bioanalytics and Agro-Metabolomics, University of Natural Resources and Life Sciences, Vienna (BOKU), Konrad Lorenz Strasse 20, 3430 Tulln an der Donau, Austria; 8 Core Facility Bioactive Molecules, Screening and Analysis, University of Natural Resources and Life Sciences, Vienna, 3430 Tulln an der Donau, Austria; 9 Present Address: DGforLife, Operations – Research and Development, Via Albert Einstein, Marcallo c.C., 20010 Milan, Italy; 10 Present Address: German Heart Center Munich, Technical University Munich, Lazarettstraße 36, 80636 Munich, Germany; 11 Present Address: Department of Biological Chemistry, Faculty of Chemistry, University of Vienna, Josef-Holaubek-Platz 2, 1090 Vienna, Austria

**Keywords:** Secondary metabolism, *Cephalotrichum*, *Doratomyces NG_p51*, Rasfonin, Transcriptome analysis, Genome analysis, In silico retrosynthesis, Biosynthetic gene cluster prediction, Natural products discovery

## Abstract

**Background:**

Fungi are important sources for bioactive compounds that find their applications in many important sectors like in the pharma-, food- or agricultural industries. In an environmental monitoring project for fungi involved in soil nitrogen cycling we also isolated *Cephalotrichum gorgonifer* (strain NG_p51). In the course of strain characterisation work we found that this strain is able to naturally produce high amounts of rasfonin, a polyketide inducing autophagy, apoptosis, necroptosis in human cell lines and showing anti-tumor activity in KRAS-dependent cancer cells.

**Results:**

In order to elucidate the biosynthetic pathway of rasfonin, the strain was genome sequenced, annotated, submitted to transcriptome analysis and genetic transformation was established. Biosynthetic gene cluster (BGC) prediction revealed the existence of 22 BGCs of which the majority was not expressed under our experimental conditions. In silico prediction revealed two BGCs with a suite of enzymes possibly involved in rasfonin biosynthesis. Experimental verification by gene-knock out of the key enzyme genes showed that one of the predicted BGCs is indeed responsible for rasfonin biosynthesis.

**Conclusions:**

This study identified a biosynthetic gene cluster containing a key-gene responsible for rasfonin production. Additionally, molecular tools were established for the non-model fungus *Cephalotrichum gorgonifer* which allows strain engineering and heterologous expression of the BGC for high rasfonin producing strains and the biosynthesis of rasfonin derivates for diverse applications.

**Supplementary Information:**

The online version contains supplementary material available at 10.1186/s40694-023-00158-x.

## Background

### Fungi as producers of bioactive substances

Filamentous fungi have the potential to produce a countless number and huge diversity of bioactive substances, also called secondary metabolites (SMs). Those substances fulfil many different functions in the ecology and lifecycles of fungi. They are produced in response to certain developmental stages, metabolic or environmental conditions and stresses, like extreme temperatures [1], osmotic stress [2], UV-radiation [3], nutrient starvation [4, 5], in defence to other organisms such as competitors [6], mating partners [7] and during host–pathogen interactions as signalling molecules or virulence factors [8]. SMs are thus molecules that are not essential for viability, but provide a selective advantage in specific situations during the life cycle of a fungal cell or colony. This is also the reason why almost all of these substances are only produced by fungi when a specific developmental, metabolic or environmental signal is present and genetically received. While some SMs can be potentially used as pharmaceuticals (i.e., lovastatin (e.g., *Pleurotus ostreatus*) [9], penicillin (*Penicillium* sp*.*) [10]), others can have detrimental effects as toxic food contaminant (i.e., aflatoxins (*Aspergillus flavus*) [11], fumonisins or deoxynivalenol (*Fusarium* sp*.*) [12, 13], etc.). Therefore, SMs are of major ecological, economical and medicinal importance.

Bioinformatic analysis suggests that fungal genomes typically hold the genetic information for dozens of so-called biosynthetic gene clusters (BGCs) that serve as blue prints for single SMs [14], and even more than 90 different BGCs in a single species have been reported [15]. This genetic diversity gives rise to an enormous variety of SMs, even not considering the numerous intermediate products that are being produced on the way [16]. This makes fungi sheer endless reservoirs for novel bioactive substances, given that there are estimated to be more than 5 million fungal species worldwide [17].

During a project that focused on the role of fungi within the nitrate assimilation process in agricultural soil, a community analysis was performed that elucidated the composition of species and the phylogenetic relationship of the genes responsible for nitrogen assimilation. For a few isolates, among them *Cephalotrichum gorgonifer* NG_p51 (previously classified as *Doratomyces sp.*), an unusual placement of the nitrate assimilation genes has been observed [18, 19]. This led to the conclusion that there had to be an, until then, unknown way of acquisition of the complete nitrate assimilation cluster including the nitrate transporter, the nitrate reductase and the nitrite reductase by horizontal gene transfer. It was proposed that the cluster was transferred from a donor at the base of the Pezizomycotina to a recipient in the Microascales, to which *C. gorgonifer belongs *[20]. Clustering of the three genes was retained, which is otherwise unusual in the Sordariomycetes. These findings prompted us to investigate also possible acquisitions of BGCs coding for SMs from other fungi to *C. gorgonifer*.

Since its first discovery in *Aspergillus nidulans* [21] it is now well established that the reason for no expression of BGCs under standard laboratory conditions is chromatin-based silencing [21–25]. We and others have thus used genetic or chemical chromatin modification to leverage the production of novel SMs in a number of fungi (e.g., treatment of *Doratomyces* sp*.* with the HDAC inhibitor valproic acid [26], deletion of a chromatin remodeler-encoding gene, *kdmB* in *Aspergillus nidulans* [27, 28], removal of heterochromatic marks in *Fusarium fujikuroi* [29], etc.). For recent reviews on methods to active silence BGCs for SMs see [25] and [30]. Using a medium throughput effort, we have tested hundreds of isolates from different environmental screening campaigns for their capacity to produce novel SMs upon chromatin activation. In the case of *C. gorgonifer* NG_p51, this led to the observation of bacterial growth inhibition and the discovery of several additionally produced compounds [26].

Further investigation in our in-house metabolite screening facility [31] revealed that *C. gorgonifer* is able to produce rasfonin, an SM initially isolated from *Talaromyces* strains and found to induce apoptosis in Ras-dependent tumor cells [32–35]. Rasfonin carries the potential of an anti-cancer pharmaceutical as it is active against several tumour types. It was shown to suppress proliferation of pancreatic cancer cells with mutated KRas and also reduced clone formation, invasion and migration of cells with mutated K-Ras. Although the exact mode of action is unknown, rasfonin was found to reduce the expression of Son of sevenless (Sos1), a nucleotide exchange factor which is involved in RAS activation [34]. In renal cancer cells, rasfonin promotes cell apoptosis and autophagy in connection with the protein Akt (protein Kinase B) which is a frequently activated oncoprotein in tumour cells [33]. Also, other studies found a connection between apoptosis and rasfonin treatment e.g., in osteosarcoma [35], renal adenocarcinoma cell lines [32] and in rasfonin-induced necroptosis [32].

Although rasfonin has the potential of being a future pharmaceutical, the biosynthetic pathway is still not known. This, however, would greatly assist further research on this SM and facilitate the generation of rasfonin over-production strains for large scale applications. We propose here the biosynthetic pathway for rasfonin in *C. gorgonifer* NG_p51 based on the presence and predicted functions of *rsf* cluster genes. Furthermore, we established state-of-the-art molecular biology methods and performed various “omics” analysis for further strain and species characterisation i.e., genome analysis, BGC prediction, transcriptome analysis, morphological description, genetic transformation and CRISPR/Cas-mediated genome editing.

## Results

### Strain characterisation of *Cephalotrichum gorgonifer* NG_p51

#### Ecology and taxonomy

**Fig. 1:**
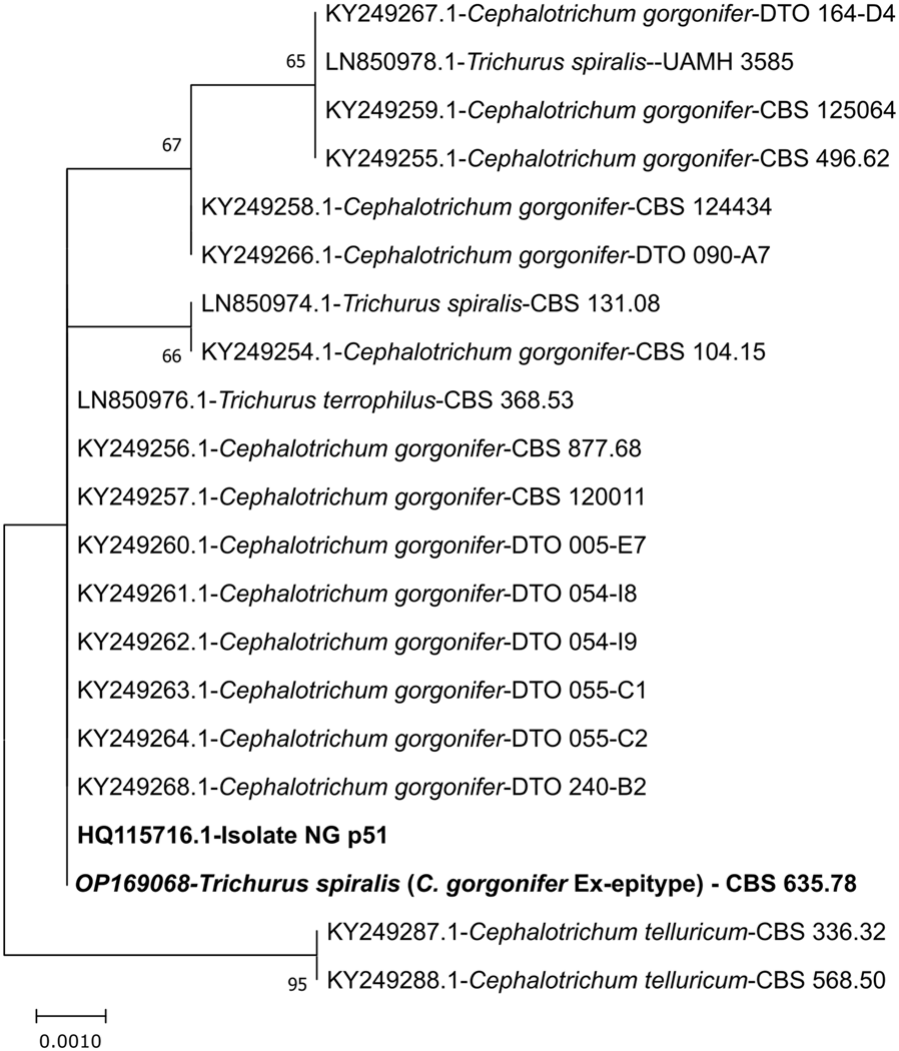
Phylogenetic tree of *Cehpalotrichum gorgonifer* isolate NG_p51. **A** Phylogenetic tree based on the ITS sequences of strain isolate NG_p51 together with several *Cephalotrichum gorgonifer* isolates and *C. telluricum* strains as closest. Tree entry structure: “accession number—genus and species—isolate identifier”. The phylogenetic analysis shows that strain isolate NG_p51 belongs to the species *Cephalotrichum gorgonifer*. The tree is drawn to scale, with branch lengths measured in the number of substitutions per site. The strain isolate (i.e. NG_p51) used for all experiments in this publication and the Ex-epitype are written in bold letters

We previously isolated the fungal strain NG_p51 and according to the former taxonomic positioning determined the genus of the strain by its ITS region as *Doratomyces* sp. (GenBank: HQ115716.1) [18]. In the meantime, the genus *Cephalotrichum* (Microascaceae, Hypocreales) has been reorganized and now contains also species formerly affiliated to the genera *Doratomyces* and *Trichurus*. No sexual morphs have been observed so far. *C. gorgonifer* is frequently found in soil, decaying plant materials, dung and on wet cellulosic materials indoors. Occasionally it has also been isolated from human hair and respiratory samples. Their temperature optimum is around 25–30 °C but also growth at 37 °C has been observed for several isolates. There are, however, no indications for human pathogenicity but data in this regard is scarce [36, 37]. We performed a phylogenetic analysis with ITS sequences (= Internal transcribed spacer regions of the rDNA and 5.8S region) of strain NG_p51 (GenBank No. HQ115716.1), together with the *C. gorgonifer* (including the Ex-epitype of *C. gorgonifer*: *Trichurus spiralis* CBS 635.-78) and *Cephalotrichum telluricum* (i.e.: closest outgroup) dataset as published by Woudenberg et al. [37]. The resulting phylogenetic tree shows that our strain clearly belongs to the species *C. gorgonifer* and that the *C. telluricum* species are clustered as an outgroup as expected (Fig. 1). We therefore designated the strain *C. gorgonifer* NG_p51*.*

#### Morphology of *C. gorgonifer* NG_p51

**Fig. 2:**
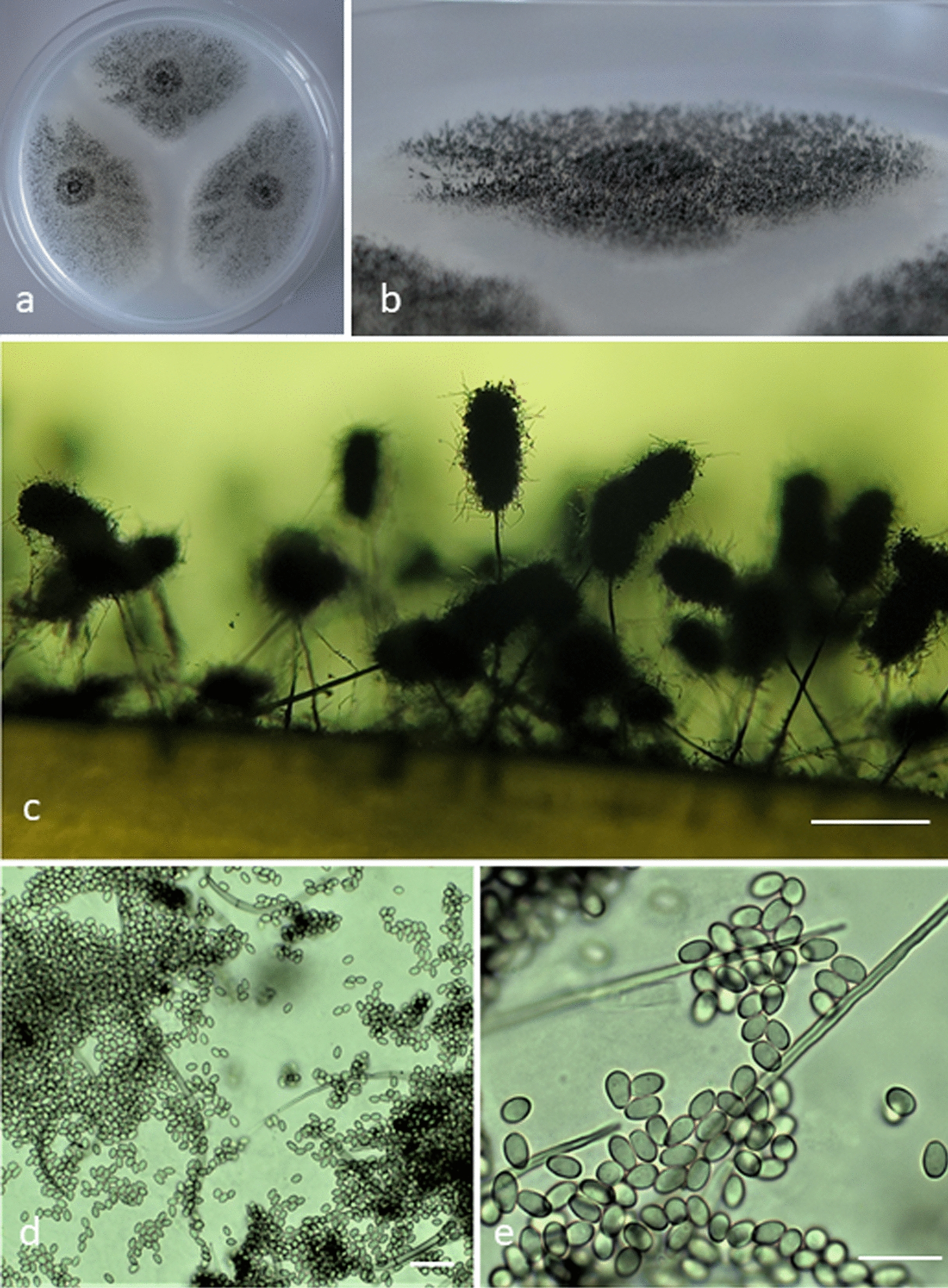
Morphologies of *Cephalotrichum gorgonife*r NG_p51. **a**, **b** Colonies on oatmeal agar (OA) after 14 d at 25 °C (Petri dish = 9 cm diam). **c** Synnemata in situ. **d, e** setae and conidia. Scale bars: c = 200 µm, d = 20 µm, e = 10 µm

Colonies of *C. gorgonifer* NG_p51 reach a diameter between 35 and 55 mm on oat meal agar (OA), potato carrot agar (PCA) and potato dextrose agar (PDA) after 14 days at 25 °C. They effuse a dark greyish black (Fig. 2a) mycelium, consisting of nearly velutinous layer of synnemata which are 1–2 mm in length (Fig. 2b) and formed by aggregation of conidiophores (the annelophores). Numerous curled sterile setae are present alongside the fertile part of the synnemata (Fig. 2c, d) and conidia are borne from ampuliform annelids which appear smooth-walled and ovoidal to broadly ellipsoidal with truncate base and rounded apex, measuring 4–6 × 3.5–4.0 µm (Fig. 2e). Phenotypic traits of the strain NG_p51 (especially presence of undulate sterile setae, greyish color of conidia *en-masse* as well as their shape and dimensions) are in perfect concordance with the species concept of *C. gorgonifer* (Bainier) Sandoival-Denis, Gené and Guarro [36], in older literature also described under a name *Trichurus spiralis* Hasselbr. by Domsch et al. [38] and Ellis [39]. Additionally, the growth on malt extract agar plates (MEA; n = 3) at 28 °C, 30 °C, 32.5 °C, 35 °C and 37 °C showed that NG_p51 behaves as published for *C. gorgonifer *[36] (Additional file 1: Fig. S10).

#### Secondary metabolite (SM) analysis of *C. gorgonifer*

Initial work with NG_p51 revealed that it is capable of suppressing growth of *Staphylococcus aureus* and two clinical methicillin-resistant isolates of *S. aureus* when the fungal strain is grown in the presence of the HDAC inhibitor valproic acid. Valproic acid treatment increased the production of seven antimicrobial compounds (cyclo-(l-proline-l-methionine), p-hydroxybenzaldehyde, cyclo-(phenylalanine-proline), indole-3-carboxylic acid, phenylacetic acid and indole-3-acetic acid) and also led to the biosynthesis of an otherwise absent compound which was identified as phenyllactic acid [26]. Also, rasfonin has been found in these screens, although the production of this bioactive compound was subsequently shown not to be dependent on valproic acid in the growth medium [31]. Initial analysis of the production conditions revealed that this metabolite is produced on liquid Aspergillus minimal medium (AMM) and detectable within 48 h of incubation (Fig. 5). Although rasfonin is a well-known bioactive compound with potential applications in cancer treatment [32–35], its biosynthetic pathway remains unknown. To decipher its production pathway, we sequenced the genome and searched for the presence of BGCs potentially coding for rasfonin production.

### Genome analysis and gene annotation

Whole genome shotgun sequencing of *C. gorgonifer* was performed by 454 pyrosequencing yielding 1.78 Gb of raw sequence data which were assembled into 48 scaffolds of overall 35.9 Mb (49.4-fold coverage, N50 of 2.07 Mb). A total of 10,469 gene models were predicted using two different GeneMark gene predictors and manual validation. The annotated ORFs account for 43.8% of the genome. The overall GC content is 55%, while the average GC content of ORFs is 60.3%. Genome sequence data and annotation was deposited in the European Nucleotide Archive (ENA) at EMBL-EBI with the project number PRJEB15373 which can be accessed online at [40].

#### Annotation of biosynthetic gene clusters (BGCs)

From one [41] to around 25 genes [42] can be involved in the biosynthesis of a single SM. In most cases these genes are coregulated and in physical vicinity to each other forming so-called BGCs [43]. Genes within these clusters can code for proteins that are responsible for the biosynthesis of the compound-defining backbone (e.g., polyketide synthase (PKS), non-ribosomal peptide synthetase (NRPS), terpene synthase (TPS)), the modification of the chemical backbone structure (e.g., cytochrome P 450, transferases, oxidoreductases, *O*-methyltransferases), the regulation of the cluster genes (global or cluster specific transcription factors (TFs)) or for the transport of synthesized metabolites (i.e., delivery to compartments within the cell or export to surrounding media) [14, 44].

For BGC annotation, antiSMASH 6.0.1 for fungi (fungiSMASH) was used with standard parameters [45]. The prediction algorithm for fungal BGCs assigned 308 genes to 24 putative BGCs. Six of them showed homology to BGCs from other species with similarities ranging from 20 to 100%. They are included in Table 1 with their respective references (only the best scoring homologous BGC is shown). Similarity in this case is defined as “percentage of genes within the closest known compound that have a significant BLAST hit to genes within the current region” (taken from antiSMASH documentation [45]). In total, ten PKSs and one type3-PKS, three NRPS-PKS hybrids, five NRPSs, six NRPS-like proteins and three putative TPSs could be assigned.

**Table 1:**
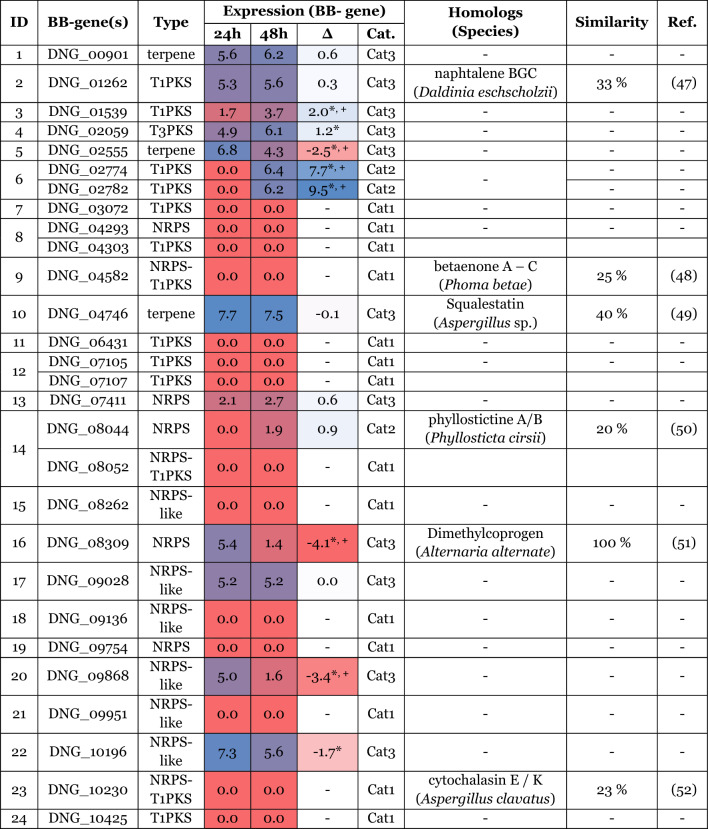
Summary of BGCs of *C. gorgonifer* NG_p51 as predicted by antiSMASH

Columns: ID: Cluster ID as assigned by antiSMASH; BB-gene(s): gene number of the key-enzyme encoding genes; type: type of key-enzyme; Expression: Expression of backbone genes at 24 h and 48 h are depicted as a 2-zone heatmap (weak expression | red → blue |strong expression), based on their RPKM value. An arbitrary log-value of 1 was considered as a biologically relevant expression threshold under which, the expression was set to zero. The column “Δ” depicts the differential expression between 24-h and 48-h samples and is visually supported by a 3-zone heatmap (down-regulated @ 48 h | blue → white → red | up-regulated @ 48 h) with zero being the turning point. A significance value of *p* < 0.05 was applied as threshold which is indicated by an Asterix near the differential expression value. If the differential expression is higher than twofold, it is indicated with an additional “+”. The Cat.: Expression Category (Cat1: Silent at 24 h and 48 h; Cat2: Expressed at either 24 h or 48 h (i.e., differentially expressed); Cat3 (Expressed at both 24 h and 48 h). Best scoring homolog BGCs in other species that were found by antiSMASH are also displayed together with their similarity in % and references

#### Analysis of candidate genes by Whole genome and transcriptome sequencing under different physiological conditions

Because rasfonin is produced not during active growth but in stationary phase cultures, we compared expression profiles between these two conditions. To obtain information about the genome-wide differential expression pattern of BGC genes we cultivated the fungus under conditions of active growth in liquid shake cultures for 24 h in AMM and compared the expression pattern with cells in stationary phase after 48 h of growth on the same medium that, at this stage, is already depleted for nutrients [5]. Of all 10,469 annotated genes, 717 genes that were predicted not to be part of a secondary metabolism pathway by antiSMASH were up-regulated at 48 h and 818 were down-regulated. From the 308 genes that are putatively involved in secondary metabolism, 39 were up-regulated at the 48-h timepoint and 40 were down-regulated. The remaining 231 genes stayed unaffected. 8855 genes were not differentially regulated between the two timepoints which are 85% of the annotated genes.

The whole transcript-dataset can be retrieved from the additional materials (Additional file 2). The whole transcriptome was uploaded at NCBIs Gene Expression Omnibus (GEO) [46] under the accession number GSE217303.

To estimate the transcriptional activity of BGCs under the chosen conditions of active growth versus stationary phase, we categorized the transcriptional status of the key-enzymes within a given BGC into three states (Table 1 Column Expression; Cat.). The expression of category 1 genes is not detectable or falls below the arbitrarily set threshold of biological relevance (see caption of Table 1) at both time-points (i.e., 14 of 29 genes). Category 2 genes (3 genes) are expressed only at one time point (i.e., either 24 h or 48 h) and the expression of category 3 genes (12 genes) is above the threshold at both 24 h and 48 h, although their expression levels may vary between the two time points (7 genes with at least a twofold difference between the time points). The individual expression levels, differential regulation between 24 and 48 h and their respective transcriptional category of all predicted key-enzyme encoding genes can be retrieved from Table 1.

### Prediction of the BGC responsible for rasfonin biosynthesis based on PKS domain structure

**Fig. 3:**
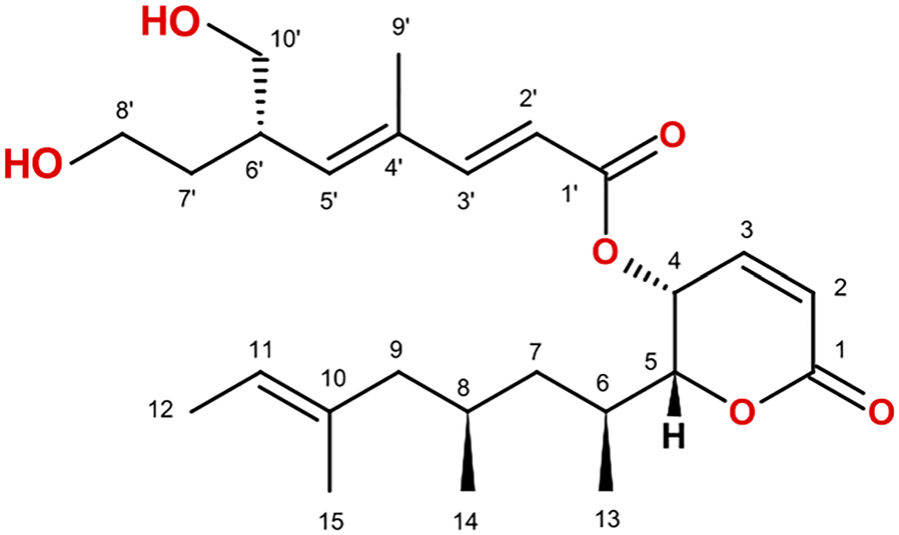
Structure of rasfonin

Rasfonin (Fig. 3) is an α-pyrone-containing natural product composed of two distinct acetate-based polyketide (PK) chains that are linked via an ester bond. The individual PK chains are likely to be derived from the condensation of four and six acetyl-CoA subunits resulting in the formation of a partially reduced and di- and trimethylated tetra- and hexaketide, respectively, exhibiting hydroxyl groups at C8′ and C10′. These chemical features suggest the involvement of two highly-reducing PKS (HR-PKSs) containing, in addition to the essential acyltransferase (AT), ketoacyl-synthase (KS), and acyl carrier protein (ACP) domains, domains involved in the reduction of the growing PK chain, i.e., a ketoreductase (KR), a dehydratase (DH) and an enoylreductase (ER). Next to this, the methyl groups at C6, C8, C10, and C4′, C6′, suggest the presence of an intrinsic C-methyltransferase (MT) domain that functions in the transfer of a methyl group from S-adenosylmethionine (SAM) to a β-ketoacyl-ACP substrate for both involved PKSs.

**Fig. 4:**
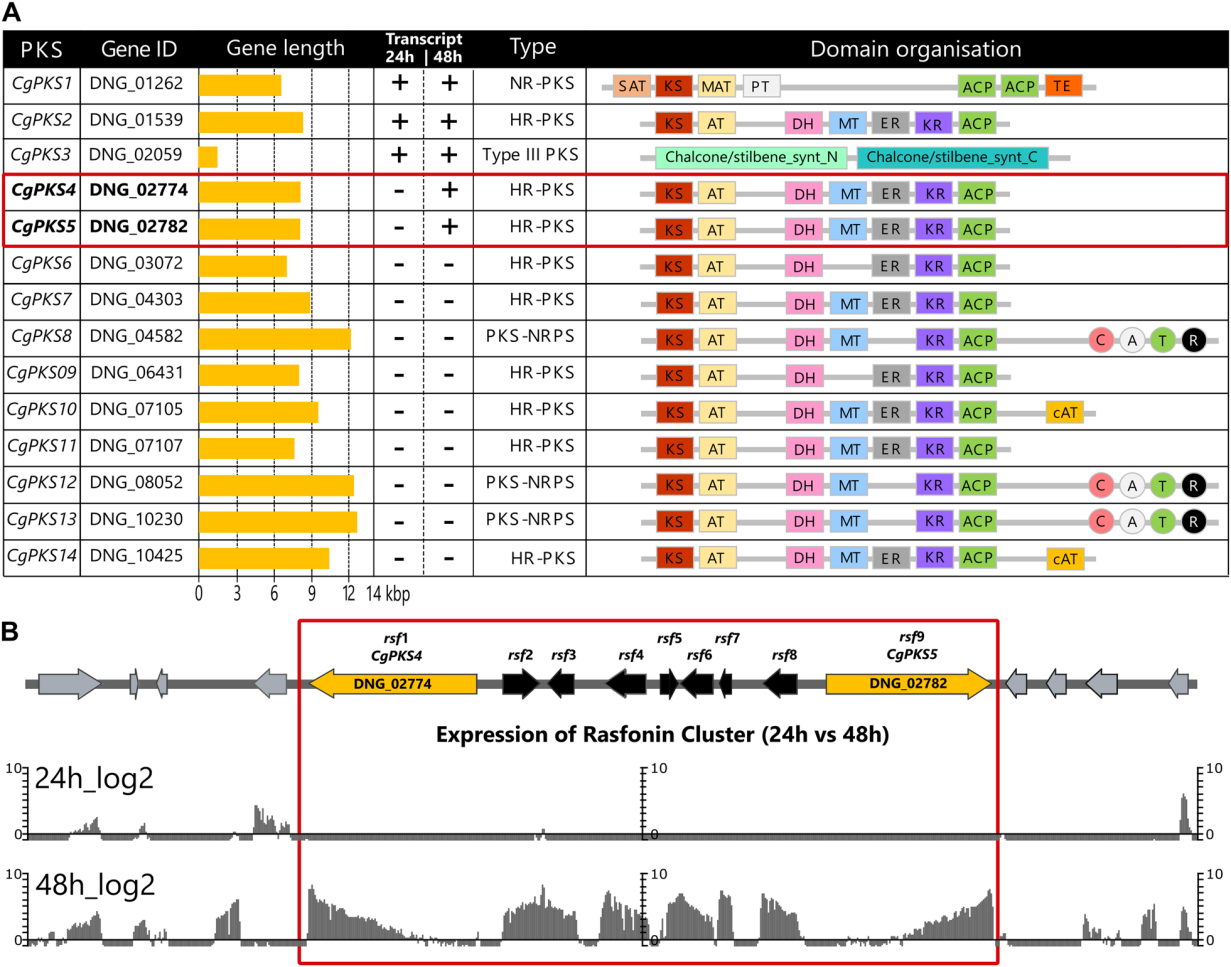
**A** Domain organization was analysed using the NCBI Conserved Domain [36], InterPro [37], SBSPKSv2 [38]; and the PKS/NRPS Analysis Web-site [39]. KS (red), keto synthase; AT (yellow), acyltransferase; DH (pink), dehydratase; MT (blue) C-methyltransferase; ER (grey) enoylreductase; KR (violet), ketoreductase; ACP (green), acyl carrier protein; cAT (orange), carnitine acyltransferase; Chalcone/stilbene_synt_N (lime-green): Chalcone/stilbene synthase N-terminal; Chalcone/stilbene_synt_C (dark-cyan): Chalcone/stilbene synthase C-terminal. The proposed PKS names are listed together with their gene number and length. The transcriptional activity at 24 h or 48 h ins indicated as “−” (No transcription at given timepoint) or “+” (transcription at given timepoint). The two PKS encoding genes that are proposed to be involved in the rasfonin synthesis (i.e. CgPKS4 and CgPKS5) are highlighted by a red box **B** The proposed BGC for rasfonin biosynthesis is depicted with transcript at 24 h and 48 h timepoint (shake flask culture, AMM). The BGC borders are depicted in red and were assigned based on the coregulation of genes. Proposed BGC gene names (rsf1 to rsf9) are depicted above gene illustration

We thus screened the genome sequence of *C. gorgonifer* for the presence of predicted PKS-encoding genes that harbour a domain composition putatively fitting the enzymatic functions necessary for rasfonin biosynthesis. Bioinformatic analysis of the 14 PKSs present in the *C. gorgonifer* genome revealed that seven HR-PKSs harbour the necessary domain architecture (Fig. 4a). Only two BGCs contain two HR-PKSs in close proximity to each other namely BGC 6 (Table 1 ID 1) with the combination of [CgPKS4 + CgPKS5] (Fig. 4a) and BGC 12 (Table 1 ID 12) with the combination [CgPKS10 + CgPKS11] (Fig. 4a). Both also encode a putative transferase, i.e., DNG_02776 and DNG_07106, respectively. To further define which of the two BGCs may be involved in rasfonin biosynthesis, we questioned our transcriptome data set and found that only the BGC with the HR-PKS pair *CgPKS4* and *CgPKS5* (Fig. 4a; Table 1 ID 6) showed expression at 48 h, the stationary phase condition conductive for rasfonin production. The other candidate gene pair *CgPKS10* and *CgPKS11* (Fig. 4a; Table 1—ID 12) was not expressed at any tested condition.

Next, we set out to analyse the extension of the CgPKS4/CgPKS5 BGC. Our transcriptome data showed a clear coregulation in expression of the genes that are positioned between *CgPKS4* and *CgPKS5*. No transcription was observed during active growth (24 h post inoculation, hpi) while expression of all putative cluster genes was significantly upregulated 48 hpi. The genes upstream of CgPKS4 and downstream of CgPKS5 were not coregulated (Fig. 4b). The GO annotations of adjacent genes do not suggest that they are involved in the synthesis of rasfonin. Thus, the BGC likely spans nine genes, i.e., DNG_02774-DNG_02782. To evaluate the contribution of the putative CgPKS4/CgPKS5 BGC to rasfonin biosynthesis, one of the PKS-encoding genes, *CgPKS4,* was chosen for targeted gene disruption.

### Experimental verification of the BGC involved in rasfonin biosynthesis

The standard method to prove the involvement of a respective candidate gene in SM biosynthesis is deletion of the coding region in the genome and the analysis if the deletion genotype is accompanied by the loss of product formation. However, so far *Cephalotrichum* sp. have not yet been genetically modified and used for reverse genetics approaches. Therefore, a transformation method had to be established with the aim to disrupt or mutate *CgPKS4* and the test by chemical analysis if rasfonin is not produced any longer in the *CgPKS*-deleted strain. To maximize the chance obtaining *CgPKS4* mutations by transformation, three different approaches were selected, i.e., a Cas9 approach (Strain Cg-Cas9-02774-1), a transformation approach mediated by *Agrobacterium tumefaciens* (Strain Cg-At-02774-1) and also a homology directed repair (HDR) approach via protoplast-mediated transformation of a linear fragment (Strain Cg-HDR-02774-1) [53, 54]. All three transformation methods were successfully implemented in our *C. gorgonifer* strain NG_p51. Cas9 and the HDR transformations were performed on cryo-stocks of *C. gorgonifer* protoplasts (see materials and methods section).

Disruption of *CgPKS4* by Cas9 resulted in the strain Cg-Cas9-02774-1. Sequencing of the locus revealed a 640 bp deletion ranging from base pair 269 (i.e.: sgRNA annealing site) to base pair 908 of the coding sequence (CDS), a mutation that additionally leads to a frameshift within the remaining CDS of the gene (Sequence at Cas9-restriction site: 5′-ACCAATGCACCCGGT | 640 bp deletion | GTTCGACCACCGCGC-3′) (see Additional file 3).

The Agrobacterium-mediated mutation resulted in the transformant Cg-At-02774-1 that has the first 2300 base pairs of *CgPKS4* (DNG_02774) replaced with the hygromycin resistance cassette (hph), again resulting in a frameshift within the CDS. The 5′-end homology region spans 1200 base-pairs upstream of the start codon (i.e. promoter region) and the 3′-homology region spans 1500 base-pairs downstream of the exchanged fragment (i.e. first 2300 base pairs of the gene body) (see Additional file 3).

The transformant Cg-HDR-02774-1 resulted from the HDR-directed approach. Here, the same replacement cassette was used as for the Agrobacterium-mediated transformation (i.e., PCR product from the “HDR-Fragment” primer pair with template pAt-DNG02774; Additional file 1: Table S1). Both strains [i.e., Cg-At-02774-1 and Cg-HDR-02774-1) were PCR tested for the successful gene replacement event (see Additional file 3: Fig. S1; Additional file 3)].

**Fig. 5:**
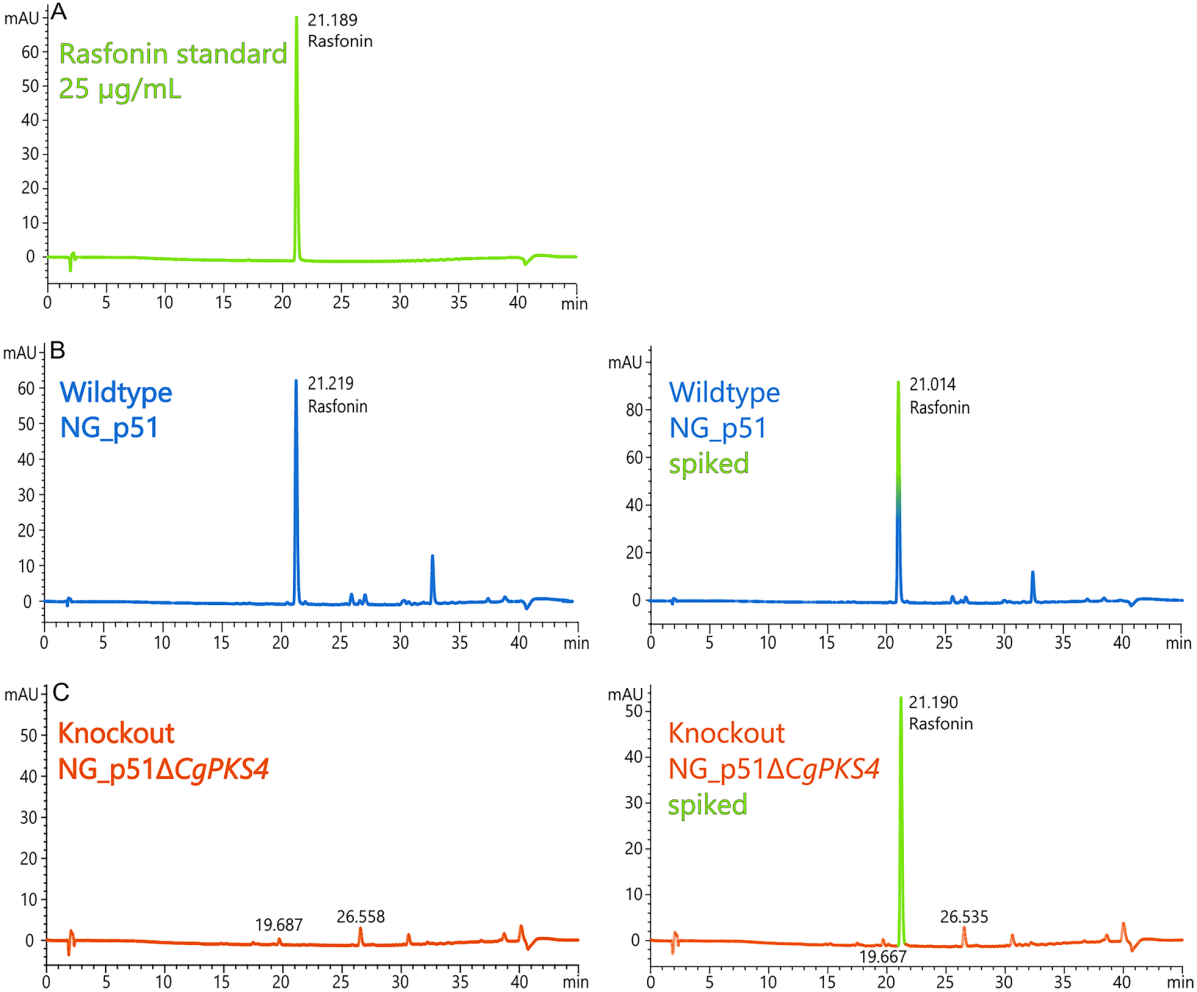
HPLC analysis of rasfonin between a 25 µg/mL rasfonin standard (green, **A**), the wildtype isolate NG_p51 (blue, **B **left) and the CgPKS4 knock-out strain NG_p51∆CgPKS4 (orange, **C **left). All 3 knock-out strains and all replicates showed the same absence of rasfonin, but only one is shown here. Spiked samples are shown on the right panel and are indicated by a green peak (NG_p51, **B **right; NG_p51∆CgPKS4, **C **right). The retention times in minutes are shown next to the peaks

Phenotypical analysis of the various *rsf1* deletion strains on MEA agar plates did not reveal any obvious morphological or growth phenotypes (data not shown). To test if the mutated target gene is responsible for the biosynthesis of rasfonin, the transformants and the recipient strain NG_p51 were cultivated in parallel on MEA agar medium. The fungal colonies (mycelia + agar media) were extracted, and extracts were analysed by high performance liquid chromatography (HPLC) for the presence of rasfonin. None of the three tested mutants showed any detectable amounts of rasfonin (Fig. 5c, left) when compared to the wildtype NG_p51 (Fig. 5b, left). A 25 µg/mL rasfonin solution produced in our laboratory and verified by NMR (see materials and methods) served as analytical standard (Fig. 5a). Next to this, we spiked the wildtype sample with our standard thereby unequivocally proofing that the peak in the wildtype chromatogram indeed is rasfonin (Fig. 5b, c, right), and that rasfonin is missing in our *CgPKS4* mutants. To verify that not even trace amounts of rasfonin are produced, that are not detected by our HPLC method, we subjected an extract from the Cg-Cas9-02774-1 strain to liquid chromatography-high resolution mass spectrometry (LC-HRMS). The results of this analysis showed no detectable amounts of rasfonin in the mutant strains (Additional file 3: Fig. S2). This verifies that CgPKS4 (from now on referred to as Rsf1) is indeed directly involved in the biosynthesis of rasfonin in *C. gorgonifer* NG_p51.

Next to *CgPKS4* (*rsf1*), eight genes are coregulated under rasfonin-producing conditions including a second PKS-encoding gene *CgPKS5* located at the end of the putative BGC. Thus, we propose that the BGC involved in rasfonin biosynthesis stretches between *CgPKS4* and *CgPKS5*, and comprises overall nine genes, *rsf1* to *rsf9* (Fig. 4b). Next to the PKSs, the BGC encodes three putative cytochrome p450s (DNG_02775: *rsf*2; DNG_02779: *rsf*6; DNG_02781: *rsf*8), a putative *O*-acyltransferase (DNG_02776: *rsf*3), a Major facilitator family1 (MFS1) transporter (DNG_02777: *rsf*4), a putative *trans*-thioesterase harbouring a FSH domain and an alpha/beta hydrolase fold (DNG_02778: *rsf*5) as well as one uncharacterised protein (DNG_02780: *rsf*7).

## Discussion

Using a combination of whole genome sequencing, bioinformatic analysis, genome-wide transcriptome analysis of cells producing versus non-producing conditions allowed us to narrow down the possible biosynthetic genes for rasfonin. These data formed the basis for prediction of enzymatic domains which are known to synthesize similar molecules. But all this prediction work in the end needs experimental verification by targeted inactivation of the proposed biosynthetic gene. For non-model fungi, the transformation-mediated inactivation of a target genes is often not trivial. Our strategy was to follow three well-known strategies to reach that goal with a high probability of success. But still, some modifications of standard protocols were necessary. Here, we developed a modified version of common protoplastation protocols to ensure a cost- and time-efficient method. The possibility to use cryo-stocks significantly increases the speed and flexibility of transformation experiments. As different fungi will need different conditions during protoplastation and post-transformation regeneration, we want to highlight a publication by Wu and co-workers [55] which compares the impact of different buffer compositions and conditions on protoplastation and regeneration efficiency. The publication assisted in the development of the protoplastation protocol for *C. gorgonifer* and will surely also be of use in case of establishment of other fungal species for the laboratory environment.

**Fig. 6:**
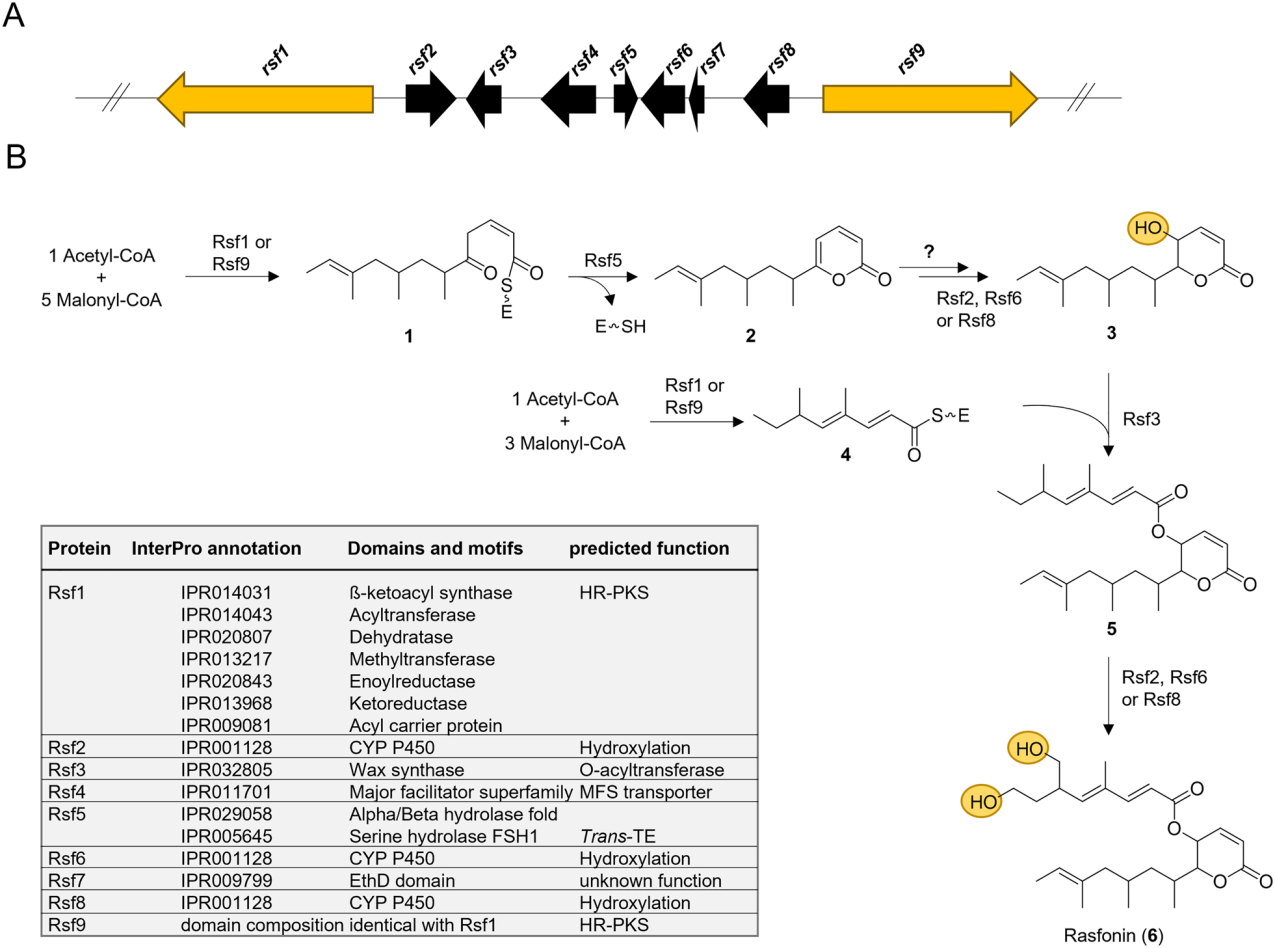
Proposed biosynthetic pathway of rasfonin. **A** The rasfonin BGC. **B** The proposed pathway of rasfonin synthesis

The biosynthesis of rasfonin is likely to be coordinated by two highly-reducing iterative type I PKSs. In general, two scenarios are possible that may yield rasfonin. In a first scenario one PKS generates a PK chain that serves as a starter unit and may be passed on to the second PKS for further extension. This mechanism has been proposed for zearalenone in *F. graminearum* [56], T-toxin in *Cochliobolus heterostrophus* [57], asperfuranone in *A. nidulans* [58] or botcinic acid in *Botrytis cinerea* [59]. In a second scenario two PKSs are involved in the assembly of two distinct PK chains that are subsequently linked via an ester bond catalysed by an acyltransferase encoded within the same BGC as it has been shown for lovastatin in *Aspergillus terreus *[60], and the related compactin in *Penicillium citrinum* [61]. Noteworthy, in rare cases one PKS is involved in the assembly of two distinct PK chains as it has been recently shown for gregatin A in *Penicillium* sp. Sh 18 [62], or fusamarin in *Fusarium mangiferae* [63], and which is likely also true for fujikurin in *Fusarium fujikuroi* [64, 65]. However, given the cluster architecture and co-regulation as well as the presence of two HR-PKS- and one putative transferase-encoding genes in the rasfonin BGC, DNG_02776 (*rsf3*), we propose the following route for rasfonin biosynthesis (Fig. 6): One HR-PKS, Rsf1 (CgPKS4) or Rsf9 (CgPKS5), is involved in the programmed assembly of the partially reduced 6,8,10-trimethylated hexaketide (**1**) by successive condensations of acetyl-CoA with 5 malonyl units, while the other assembles the 4′,6′-dimethylated tetraketide (**4**). Noteworthy, none of the HR-PKSs harbours a release domain, but the presence of Rsf5, a predicted thioesterase (TE) with similarity to LovG from the lovastatin cluster [66], suggests that Rsf5 may function in the release of the hexaketide. Similar *trans*-TEs have been shown to facilitate off-loading in the case of fusarielin biosynthesis in *F. graminearum* [67], and fusamarin biosynthesis in *F. mangiferae* [63]. The PK chain may be released either by TE-mediated hydrolysis followed by the spontaneous intramolecular cyclisation accompanied by dehydration yielding **2**, or the cyclisation happens directly through TE-catalysed pyrone formation as it has been shown for the TE-domain of CTB1 involved in cercosporin biosynthesis in *Cercospora* sp. [68]. **2** is then further modified to yield **3** by hydrogenation and subsequent hydroxylation of C4. The latter step is likely catalysed by one of the cytochrome p450s encoded within the BGC, Rsf2, Rsf6 or Rsf8. Next, Rsf3, encoding a putative O-acyltransferase with similarity to LovD [69] captures **4**, followed by the transacylation of **3** resulting in the formation of **5**, that is further hydroxylated at C8′ and C10′ likely catalysed by one or both of the remaining cytochrome p450s encoded in the BGC, yielding the final product rasfonin (**6**).

In fungi, the true physiological or ecological function of rasfonin remains obscure. No strong antimicrobial activity has been detected so far. Available data from experiments with cancer cell lines suggest a modulation of Ras-signalling pathways by rasfonin, which can suppress proliferation of certain cell lines [34]. As a colonizer of cellulosic plant litter, *C. gorgonifer* would encounter numerous other eukaryotes, where alterations of their developmental program through modulation of Ras signalling could be potentially advantageous for the producing organism. However, other eukaryotic cells tested by us, such as *Saccharomyces cerevisiae*, seem highly resistant to this compound (our unpublished preliminary observations). The availability of KO strains without detectable rasfonin production will allow to test hypotheses on rasfonin function in the natural environment.

An interesting preliminary observation of our study was that the metabolite does not seem to be actively exported into the surrounding of the cell but stays within or attached to the mycelium in a fungal culture (unpublished preliminary observation). Although there is an expressed gene within the BGC that codes for a transporter, computational analysis of the predicted protein sequence by MULocDeep suggests that the transporter is targeted to a lysosome-like membrane [70]. This indicates that an intermediate or the final compound is stored within extra compartments like it is the case with fungal toxisomes [71, 72]. Of course, some exporter, encoded by a gene residing externally of the rasfonin BGC, might be responsible for export, but this topic needs further investigation.

## Conclusions

This work provided the tools and methods for working efficiently with the rasfonin producing strain *Cephalotrichum gorgonifer NG_p51* in a laboratory environment. Thorough analysis of the genome and different transcriptomes in combination with metabolic and taxonomic studies have allowed us to identify a BGC that is responsible for the biosynthesis of rasfonin. This now enables further train and pathway engineering with the aim to develop a high yield production strain of this pharmaceutically relevant compound. The establishment of three different transformation methods and the development of an adapted protocol for protoplast and protoplast-cryo-stock preparation of *C. gorgonifer* NG_p51 surely helps to speed up molecular genetics work with this fungus and will also help with establishing such methods in other fungal species promoting the identification of compounds encoded by so far orphan BGCs.

## Materials and methods

### Phylogenetic tree: evolutionary analysis by maximum likelihood method

ITS sequences (For accession numbers see Fig. 1) were aligned with the program MEGAX: Molecular Evolutionary Genetics Analysis across computing platforms (Version 10.2.6) [73] with the ClustalW algorithm (standard parameters) [74]. After alignment, sequences were trimmed to equal length for the construction of the phylogenetic tree.

The tree with the highest log likelihood (− 808.77) is shown. The percentage of trees in which the associated taxa clustered together is shown next to the branches. Initial tree(s) for the heuristic search were obtained automatically by applying Neighbor-Join and BioNJ algorithms to a matrix of pairwise distances estimated using the Tamura-Nei model [75], and then selecting the topology with superior log likelihood value. This analysis involved 21 nucleotide sequences. There was a total of 557 positions in the final dataset. Evolutionary analyses were also conducted in MEGA X. Bootstrapping was performed 500 times.

### Morphological analysis of strain NG_p51

For phenotypic characterization, the strain *Cephalotrichum gorgonifer* NG_p51 was transferred on Potato Dextrose Agar (PDA, Fluka), Malt Extract Agar (MEA, Merck) and Oatmeal Agar (OA) as described by [76] and incubated for 14 days in the dark at 25 °C. For comparative description of the macroscopic and microscopic characteristics, OA was used according to [36], and additionally compared to [38, 39] where the fungus is described and illustrated under the name *Trichurus spiralis* (current name: *Cephalotrichum gorgonifer* [36]). The photomicrographs were taken using a Motic BA 310 microscope with Motic Image Plus 3.0 software. Lactophenol blue was used as a mounting medium for microphotography. Photographs of the colonies were taken with a Sony DSC-RX100.

### Growth phenotype at different temperatures

For determining the growth morphology under different temperatures, spores of strain NG_p51 were grown by plating them from cyro-stocks (50% Tween-20 0.1% spore suspension and 50% glycerol 50% (w/v)) on MEA plates at 30 °C for 4 days. Spores were harvested by scraping off the agar with a spatula and 0.1% Tween solution. The spore suspension was then filtered through glass wool filled tips and a 1/1000 dilution was prepared (3 subsequent 1/100 dilutions; 100µL spore solution and 900 µL Tween 0.1%) were then counted in a counting chamber (Neubauer improved; PC72.1—Carl Roth) and a dilution with a spore density of 100 spores/µL (Tween-20 0.1%) was prepared. 10 µL of the spore solution was then applied in the middle of a MEA plate and 3 plates each were incubated at 28 °C/30 °C/32.5 °C/35 °C/37 °C for 4 days. The growth was measured in mm diameter.

### Genome sequencing and gene prediction

The genome sequencing and assembly of *Cephalotrichum gorgonifer* was performed using a whole-genome shotgun approach that explored paired-end 454 (LGC Genomics GmbH, Berlin, Germany). Using Newbler v2.6. (Roche (2010) 454 Sequencing System Software Manual, v 2.5.3: Part C—GS De Novo Assembler, GS Reference Mapper, SFF Tools, 454. Life Sciences Corp, A Roche Company, Branford) shotgun and 8 kb-PE library reads were assembled into 702 contigs. Pre-assembled contigs were combined into 48 scaffolds using the SSPACE algorithm [77]. To predict genes on the scaffolds two ab initio gene predictor algorithms were applied, GeneMark-S and GenMark-ES version 2.3 [78]. Gene models differently predicted by the algorithms were manually curated. The genome was analysed using the PEDANT system [79] to allow comparative feature analysis.

### Transcriptome: RNA sequencing and analysis

Total RNA extraction samples (24 h/48 h; 6 µg each) were transferred to Vienna Biocenter Core Facilities [80] for library preparation and Illumina high throughput sequencing using poly-A enrichment kit (NEB) and Nextera Library prep kit. 50 bp single end sequencing was performed using a HiSeq v4 Illumina sequencer. Obtained sequences were de-multiplexed, quality controlled, filtered using trimmomatic 0.36 [81] and mapped on the *Cephalotrichum gorgonifer* NG_p51 genome assembly (ENA project number PRJEB15373). Mapping was performed using BWA [82]. Mapping data were processed using samtools [83] and duplicate reads were removed using Picard MarkDuplicates accessible at [84]. Reverse transcripts were counted using python script HTSeq [85]. Normalization and statistics were done using R/Bioconductor and the limma and edgeR packages, using mean–variance weighting (voom) and TMM normalisation [86] and robust linear model fitting. A significance cut-off of *p* < 0.05 and an absolute difference of 1 (i.e. twofold regulation) was applied for analysis. Transcription levels are log2 read counts per kilobase of exon per million library reads (RPKM). For trace graphs transcript coverage was calculated using bedtools genomecov [87, 88] for each bp normalized to sequencing library sizes as log2 scaled counts per million reads (CPM). All data are available at NCBI GEO under the accession number GSE217303.

### Molecular biology methods

For gDNA extraction, *Cephalotrichum gorgonifer* colonies were inoculated in 1 mL liquid Czapek DOX media with 10 mM NO_3_ in Lysing Matrix A tubes (MP Biomedicals; Irvine, CA; Art. No. SKU 116910100) for 24–48 h at 30 degrees and 180 rpm. After incubation, the cultures were centrifuged at 20,238 rcf in an Eppendorf 5424 centrifuge. The supernatant was removed and 1 mL CENIS lysis buffer (200 mM TRIS–HCl pH 8.5, 250 mM NaCl, 25 mM EDTA, 0.5% SDS) was added. A FastPrep-24™ ribolyzer (MP Biomedicals; Irvine, CA; Art. No. SKU116004500) was then used to homogenize the samples (20 s, 6.0 m/s) and the gDNA extraction according to CENIS was conducted [89]. The dried gDNA was dissolved in 100 µL of dH_2_O and heated up to 65 °C for 10 min. 1 µL was taken for PCR.

Polymerase Chain Reaction (PCR) was either performed with the Q5^®^-polymerase from New England Biolabs (New England Biolabs; Art. No. M0491L) for plasmid cloning, or with the GoTaq^®^ Green Master Mix (Promega; Madison, WI, USA; Art. No. M7845) if used for diagnostic PCRs (i.e. strain and plasmid verification). PCR was performed according to the respective manual. Plasmid preparations were performed with Genejet^®^ Plasmid Miniprep Kit (Thermo Scientific™, Art. No. K0503). All information (primer pair names and sequences, amplicon names and templates) can be retrieved from Additional file 3: Table S1.

Plasmid construction was performed with either NEBBuilder^®^ HiFi DNA Assembly Master Mix (New England Biolabs (NEB); Art. No. E2621S) or by yeast recombinational cloning (YRC) [90]. All PCR fragments were purified with the Monarch PCR & DNA Cleanup Kit (NEB; Art. No. T1030L), measured at a NanoDrop™ spectrophotometer (Thermo Fisher Scientific™; Art. No. ND-2000C). All reactions were conducted according to the manufacturer’s manual. All resulting plasmids were verified by sequencing (“Ready2Run” by LGC Genomics GmbH; Berlin, Germany). The plasmids can be retrieved in GenBank format from the additional files (Additional file 4–Additional file 7). A summary of all plasmids that have been constructed during this study and their main elements is contained in Additional file 1: Table S2.

For construction of the *Agrobacterium tumefaciens* transformation plasmid pAt-DNG02774, plasmid pCBRS [91] and gDNA from *C. gorgonifer* strain NG_p51 were taken as templates for PCR with Q5 polymerase. Q5 products Q5_At_02774_1 to Q5_At_02774_5 (Additional file 3: Table S1) were used for YRC. The correct assembly was verified by restriction digest of the resulting plasmid with MlsI from Thermo Scientific. The plasmid contains the features needed for agrobacterium mediated transformation and an exchange cassette which replaces the first 2300 base-pairs of gene DNG_02774 (i.e.: CgPKS4) with the hph marker.

For plasmid pCas9-DNG02774-sgRNA5 which was used for the Cas9 mediated mutation of DNG_02774, the two precursor plasmids, pCas9-scaff and pCas9-hph were constructed.pCas9-scaff was assembled by using HiFi and PCR fragments Q5-Cas9-Scaff-1 to Q5-Cas9-Scaff-7 (Additional file 1: Table S1). Templates for the PCRs were the plasmid pFC331 [54], gDNA from an *Aspergillus fumigatus* wild-type isolate (in-house strain collection AIT-#1232), gDNA from *Aspergillus nidulans WIM 126 *[92]*,* plasmid psgRNA [93], plasmid pCSN44 [94] and the plasmid pRS426 [95].

For the construction of pCas9-hph, plasmid pCas9-scaff was cut with Eco72I (Thermo Scientific™; Art. No. ER0361) and used for HiFi assembly with PCR fragment Q5-Cas9-hph that contains the hph marker (Additional file 1: Table S1).

For construction of plasmid pCas9-DNG02774-sgRNA5, plasmid pCas9-hph was cut with Bsp1407I (Thermo Scientific™; Art. No. ER0932), and assembled together with an sgRNA oligonucleotide with the sequence 5′-gtaccagacgaatctacacaCAAGGACTGATGAGTCCGTGAGGACGAAACGAGTAAGCTCGTCTCCTTGACGAAGTAGCCACCgttttagagctagaaatagc-3′. The sgRNA has the sequence: 5′-TCCTTGACGAAGTAGCCACC | GGG (PAM)-3′ and anneals around base pair 275 downstream of the start codon of CgPKS4 (DNG_02774).

### Strain construction

All fungal strains used during this study are listed in Additional file 1: Table S3.

### Agrobacterium transformation

Strain Cg-At-02774-1 was generated by disruption of CgPKS4 by agrobacterium mediated transformation with plasmid pAt-DNG02774. The transformation was conducted according to [96, 97] with the deviation that fungal cultures were pre-grown on CM-agar plates and that for co-cultivation of bacteria and fungi, not cellophane or agar-blocks were used, but 200µL of a 1:1 mixture of a fungal spore solution (1 × 10^6^ spores/mL in Tween-20 0.1%) and an induced *Agrobacterium* culture broth (*Agrobacterium tumefaciens* carrying pAt-DNG02774) were plated on 10 MoserIND petri dishes each. After 4 days of incubation at 20 °C, the plates were overlain with 15 mL of ~ 40 °C warm selection media (YES + hygromycinB + cefotaxim) and incubated for further 3 days at 30 °C before picking transformants. After purification via single spore isolation, the proper insertion of the replacement cassette was verified by Q5^®^-PCR with primer pair At-HDR_Diag (Additional file 1: Table S1) which amplifies the region that spans from outside of the homology regions and across the whole insert. The proper integration was assessed via size comparison of amplicons of the recipient strain (i.e. NG_p51) and the mutant on an agarose gel.

### Disruption of *CgPKS4* by protoplast transformation (Cas9 and HDR)

For the disruption of gene DNG_02774 by Cas9 (i.e. Cg-Cas9-02774-1) or by a linear fragment (HDR; i.e. Cg-HDR-02774-1), 10 µg plasmid pCas9-DNG02774-sgRNA5 respectively 72 µL of an un-purified Q5^®^-PCR product (i.e. “HDR-fragment” which is the exact same replacement cassette as within pAt-DNG02774; (Additional file 1: Table S1) was transformed via a modified protoplast transformation protocol into strain NG_p51 as described below. In case of the Cas9 approach, successful disruption was verified by amplification of the promoter and 5′ end of the gene DNG_02774 (~ 2 kb) with primer pair Cas9-Diag (Additional file 1: Table S1) which showed a 600 base-pair deletion, starting at the sgRNA annealing site. The deletion was further confirmed by sequencing with primer 5′-ATCACTCCCATAGCCGTCATCG3-3′ at the LGC genomics sequencing facility (Berlin, Germany). In case of the HDR approach, the whole replacement cassette was amplified from outside of the homology regions, exactly as described above for the verification of the agrobacterium transformation (i.e. Cg-At-02774-1).

### Modified protoplastation and transformation protocol for *C. gorgonifer*

For protoplast preparation, spores were harvested with Tween^®^ 20 0.1% from a yeast extract sucrose (YES) agar plate and filtered through a glass wool tip. 6 mL of YES-broth in an eprouvette were inoculated with a spore density of 4 × 10^6^ spores per mL media and incubated for 18 h at 30 °C at 180 rpm. The culture was transferred into a falcon tube and centrifuged for 5 min at 4 °C and 2218 rcf in a 5810 R Eppendorf centrifuge with swingout rotor. The supernatant was discarded and 1 mL of Lysing solution was prepared: 12.5 mg Driselase from Basidiomycetes (Sigma-Aldrich; Art. No. D9515), 1 mg Chitinase, from *Streptomyces griseus* (Sigma-Aldrich; Art. No. C6137), 25 mg Lysing enzymes from *Trichoderma harzianum* (Sigma-Aldrich; Art. No. L1412) in 1 mL of “OsmoStock” solution (40 mM/L Citric acid, 1.1 mM/L MgSO_4_; set pH to 5.9 with 3 mol/L NaOH). The Lysing solution was added to the mycelium. The mixture was resuspended and transferred into 2 mL tubes and put into an “IKA KS4000 I control” shaker at 30 °C at 110 rpm for 5 h. After visual check of protoplasts, the mixture was centrifuged at 4 °C for 10 min at 6000 rcf in an Eppendorf 5424R centrifuge and as much intermediate layer (clean protoplasts on top, protoplasts and cell debris at bottom) as possible was removed. Protoplasts count was determined in a counting chamber (Neubauer improved; PC72.1—Carl Roth, Karlsruhe).

For preparation of protoplast cryo-stocks, the solution was set to 4 × 10^8^ protoplasts per mL with OsmoStock solution and the same amount of 50% glycerol was added and mixed. Aliquots of 90 µL were then slowly frozen in 1.5 mL Eppendorf tubes within two styro-foam boxes at − 80 °C. Each tube holds protoplasts for 3 transformations (i.e.: 6 × 10^6^ protoplasts per transformation).

For protoplast transformation, one protoplast cryo-stock was thawed on ice. 6 × 10^6^ protoplasts (i.e. 30 µL) were taken and adjusted to 200 µL with ice-cold TN1 solution (i.e. modified STC buffer solution: Sorbitol 0.8 mol/L, CaCl_2_ 50 mM/L, Tris–HCl pH 8 10 mM/L) [98]. 10 µg plasmid DNA (i.e.: pCas9-DNG02774-sgRNA5) or 72 µL of non-purified Q5-PCR product HDR-fragment (Additional file 1: Table S1) and 50 µL of ice-cold TN2 (= TN1 with PEG 4000 40% (w/v)) solution were added and mixed gently per pipetting. The whole mixture was left on ice. After 30 min, 1 mL of TN2 solution was added, gently mixed and left at room temperature for 5 min. The mixture was then added to 45 mL of ~ 40 °C Bottom Agarose (AMM [99] + 0.8 mol/L Sucrose + 10 mM/L NaNO_3_ + 0.8% w/v low melt agarose (Carl Roth; Art. No. 6351.2) and equally distributed into 3 petri dishes. After 2 h at room temperature, 15 mL of 45 °C Top agarose (same recipe as Bottom Agarose but 1.5% (w/v) low melt agarose and 0.05 mg/mL hygromycin B (Merck Millipore; Art. No. US1400052; potency: 1140 µg/mg) was added to each plate. After solidification, plates were incubated at 30 °C until colonies appeared on the surface (~ 7 days). Colonies were picked and two rounds of single spore isolation (strike out—pick—grow until sporulation) were conducted on Aspergillus complete media agar plates (CM by DR. C. F. Robert [99]) with hygromycinB before preparing spore cryo-stocks.

### Spore-cryo-stock preparation

The fungus was grown on a CM plate and incubated for 6 days at 30 °C. A spore suspension with Tween-20 0.1% (v/v) was prepared, mixed with a 50% (w/v) glycerol solution at a ratio of 1:1 and 5 × 750 µL aliquots were frozen at − 80 °C.

### Fermentation and extraction for rasfonin standard preparation

Fermentation conditions, isolation and structural elucidation was carried out according to Labuda et al. [100]. The fungal spore suspension (5.0 × 10^6^ spores/mL) was obtained after 7 days cultivation of the fungus (*Cephalotrichum gorgonifer* NG_p51) on a potato dextrose agar (PDA, VWR Chemicals, Austria). Five colony plugs were cut (each ca 1 × 1 cm) and thoroughly mixed (on vortex for 2 min) with 30 mL of physiological solution (0.9% NaCl) in a sterile, 50 mL Falcon tube. In total, 2 kg of parboiled rice was used as a production substrate. Briefly, a total of 100 g of rice mixed with 100 mL deionized water was let soaked for ca one hour in 1000 mL capacity Erlenmeyer flasks, and then sterilized under standard autoclaving conditions (20 min, 121 °C, under pressure). Each flask (n = 20) was inoculated with 100 µL of spore suspension at the central part of the surface. The flasks were cultivated in perforated plastic bags for 28 days at 30 °C in the dark. At the end of the cultivation, the rice cultures were checked for purity, cut into small pieces and the whole content of the plates (fungal colonies with rice-substrate) was harvested into a 5 L glass flask. No heating deactivation nor drying was performed. The material was then mixed with 2 L of acetonitrile (ACN, Honeywell, Seelze, Germany). After vigorous stirring for 2 min in three subsequent steps (with ca 20 min in between), the mixture was filtered through a steel sieve in order to separate the solid particles (fungus and medium). The remaining residual water (generated by condensation of the water on plates during fungal growth) was removed by the addition of 10 g of anhydrous sodium sulphate. The organic phase was then filtered through a filter paper (270 mm i.d., Macherey–Nagel, Düren, Germany) and concentrated under reduced pressure at 45 °C (Büchi Rotavapor R-114, Flawil, Switzerland). The whole extraction procedure was repeated twice and yielded 3.5 g of crude culture extract.

### Isolation of secondary metabolites for rasfonin standard preparation

The crude extract was purified by reversed-phase silica gel vacuum flash chromatography (Interchim, puriFlash^®^450, Montlucon Cedex, France), using three consecutive Interchim puriFlash^®^ 32 g silica IR-50C18-F0025 flash columns (particle size: 50 µm). The columns were eluted with a binary solvent gradient (solvent A: H_2_O, solvent B: ACN). The starting linear gradient from 10% B to 27% B during 25 min at a flow rate 15 mL/min was followed by an isocratic gradient at 52% B for 10 min. Then a linear gradient from 52 to 66% B over 7 min was applied at the same flow rate and finally the column was washed starting with 100% B for 10 min followed by 100% A for 10 min at a flow rate 15–30 mL/min. UV 254 nm and UV scan 200–400 nm mode were used for detection and final separation of 7 main fractions (F1-F7), which were consequently concentrated under reduced pressure at 45 °C. The target compound was found in fraction F5 (23–27 R_t_, yield: 370 mg). It was resolved in a solvent mix (1:1:1; ACN/CH_3_OH/H_2_O) and further purified by an Agilent 1260 Infinity preparative HPLC (USA) on a reversed phase column Gemini NX C-18 (21.20 × 150 mm, 5 µm, 110 Å). Gradient starting with 30% ACN and 70% H_2_O up to 90% ACN in 10 min (total time 34 min) and a flow rate of 25 mL/min. Four fractions (pF1–pF4, time slice each 0.5 min) were collected, of which pF4 contained the target-rasfonin. Yield of rasfonin in pF4 (t_R_ 17.85–18.70 min) after one stage of prep HPLC was 250 mg. For purity check an Agilent 1200 system was used with the same stationary phase (Gemini 5 µm NX-C18 110 Å, 150 × 2 mm) and gradient program at a flow rate 0.3 mL/min. Analytical chromatogram of purified rasfonin and its UV spectrum is presented on Additional file 1: Fig. S3. The identity of rasfonin was furthermore verified by NMR analysis (see below).

### LC-HRMS

LC-HRMS measurements were carried out with a Vanquish UHPLC system coupled to a QExactive HF Orbitrap mass spectrometer (Thermo Fisher Scientific). For chromatographic separation a C18 reversed phase column (X-Bridge, 150 × 2.1 mm id, 3.5 µm particle size, Waters) was used with gradient elution applying H2O + 0.1% FA (eluent A) and methanol + 0.1% FA (eluents B) as eluents with a constant flow rate of 0.25 mL/min. After an initial minute with constant 10% eluent B, a linear increase towards 100% B was applied in 9 min followed by 3 constant minutes of 100% B. 7 min of re-equilibration with 10% B completed the method with a total length of 20 min. Ionisation was carried out in fast polarity switching mode and full scan mass spectra were recorded with a mass range of m/z 100–1000 and a resolution of 120 000 at *m/z* 200 (FWHM). MS/MS measurements for [M + H]^+^ of Rasfonin were carried out in a data dependant method using an inclusion list. The selected ions were fragmented with stepped normalized collision energy (25, 35, 45 eV) and recorded with a resolution of 15 000 at *m/z* 200 (FWHM) at mass ranges *m/z* 50–460. For compound identification the in-house purified reference standard of Rasfonin (see above) was dissolved and diluted to a concentration of 1 mg/L and measured with the described method within the same sequence as all biological samples.

### NMR

All NMR spectra were recorded on a Bruker Avance II 400 (resonance frequencies 400.13 MHz for ^1^H and 100.63 MHz for ^13^C) equipped with a 5 mm N_2_-cooled cryoprobe (Prodigy) with z–gradients at room temperature with standard Bruker pulse programs. The sample was dissolved in 0.6 mL of CDCl_3_ (99.8% D). Chemical shifts are given in ppm, referenced to residual solvent signals (7.26 ppm for ^1^H, 77.0 ppm for ^13^C). ^1^H NMR data were collected with 32 k complex data points and apodised with a Gaussian window function (lb =  − 0.3 Hz and gb = 0.3 Hz) prior to Fourier transformation. ^13^C spectrum with WALTZ16 ^1^H decoupling was acquired using 64 k data points. Signal-to-noise enhancement was achieved by multiplication of the FID with an exponential window function (lb = 1 Hz). All two-dimensional experiments were performed with 1 k × 256 data points, while the number of transients (2–16 scans) and the sweep widths were optimized individually. HSQC experiment was acquired using adiabatic pulse for inversion of ^13^C and GARP-sequence for broadband ^13^C-decoupling, optimized for ^1^J(CH) = 145 Hz. For the NOESY spectrum a mixing time of 0.5 s was used.

### Structure determination of rasfonin (Fig. 3) standard by NMR

The isolated compound showed a [M + H]^+^ peak at *m/z* = 435.2697 1229 (calcd 435.2741 for C_25_H_39_O_6_) in the ESI spectrum which is in accordance with the expected molecular formula of C_25_H_38_O_6_. The ^1^H and ^13^C nmr spectra revealed the presence of three doubletic methyl groups at δ_H_/δ_C_ 1.54/13.33, 1.15/18.85, 0.78/20.59 ppm and two singlet methyl groups at 1.52/15.53, and 1.83/12.59 ppm, respectively. In addition, 6 olefinic protons could be detected. Two pairs of them are part from delocalized double bond systems due to conjugation to carboxylic carbons C-1 and C-1′: H-2/C-2 and H-3/C-3 showed signals at δ_H_/δ_C_ 6.21/124.93 and 7.04/140.58 ppm, whereas the system 2′ and 3′ resonated at δ_H_/δ_C_ 5.81/115.01 and 7.34/150.96 ppm, respectively. Furthermore, two oxygenated methines and two oxygenated methylene groups were identified in the nmr spectra. Detailed analysis of all two-dimensional nmr spectra proved the presence of rasfonin for the isolated compound. All assigned chemical shifts (Additional file 1: Table S4, Figs. S4–S9) are in agreement with those already reported for rasfonin by Fujimoto et al. [101].

### Rasfonin analysis of *CgPKS4* knock-out strains

For rasfonin analysis of *CgPKS4*-mutants, triplicates of each knock-out strain and the wildtype NG_p51 were cultivated. Fungal spores were plated on YES-agar plates supplemented with 100 mM NO_3_ and cultivated at 30 °C for 7 days. The culture + agar was then cut into ~ 1 cm^2^ pieces, frozen in liquid nitrogen and lyophilised in a Christ Alpha 2–4 LSCbasic lyophilizer. The freeze-dried material was ground in a Retsch MIXER MILL MM 400 ball mill in a liquid nitrogen cooled 50 mL grinding jar with one 20 mm stainless steel grinding ball per jar at 25 Hz for 60 s. 0.1 g of the ground material were extracted with 1.5 mL ACN with 0.1% FA acid on an IKA Vibrax VXR basic shaker at 1500 rpm for 1 h at room temperature. The slurry was then centrifuged for 20 min at 20,238 rcf and at room temperature in an Eppendorf 5424 centrifuge. The supernatant was then filtrated through a 0.22 µM PTFE syringe filter, and the extract was reduced in a prewarmed (45 °C) Eppendorf concentrator plus for 30 min at V-HV settings. The reduced extracts were refilled to 300 µL with ACN and filtered again to remove precipitate that formed during concentration. The extracts were directly measured in via HPLC and LC-HRMS.

### Measurement of knock-out-mutants via HPLC

Samples were analyzed with an Agilent 1200 system with a reversed phase column Phenomenex Gemini NX C-18 (2 × 150 mm, 5 µm, 110 Å). Injection volume of samples was 20 µL. The flow rate was 0.3 mL/min with a binary gradient of A from 15% (ACN + 0.1% FA) to 95% in the time interval 2–31 min (Solvent B: MQ-H_2_O + 0.1% FA). Rasfonin was detected with an Agilent G1315C DAD detector at 280 nm. Elution time of rasfonin was at around 21.1 min. For reference measurement and sample spiking, a 25 µg/mL rasfonin standard produced by our laboratory (see above) was used.

## Supplementary Information

**Additional file 1. Table S1**: Information about oligonucleotides, strain verification, LC-HRMS analysis of KO strains, plasmid information, strain information, UV spectrum of rasfonin, nmr data of rasfoninm, Growth of *C. gorgonifer* at different temperatures.

**Additional file 2**. Transcriptome dataset and analysis of *C. gorgonifer* NG_p51 at 24h and 48h in AMM liquid media.

**Additional file 3**. Genbank file with the rasfonin BGC and surrounding genetic locus, information on *CgPKS4* disruptions of KO strains.

**Additional file 4**. Plasmid map pAt_DNG02774 in genbank format.

**Additional file 5**. Plasmid map pCas9-DNG02774-sgRNA5 in genbank format.

**Additional file 6**. Plasmid map pCas9-hph in genbank format.

**Additional file 7**. Plasmid map pCas9-scaff in genbank format.

## Data Availability

The datasets that were generated during this study are accessible at following repositories: Genome and Gene annotation of *C. gorgonifer* NG_p51: European Nucleotide Archive (ENA) at EMBL-EBI. Project number PRJEB15373 which can be accessed at http://www.ebi.ac.uk/ena/data/view/ONZQ01000001-ONZQ01000048 [40]. Transcriptome data of *C. gorgonifer* NG_p51 at 24 h and 48 h: The data discussed in this publication have been deposited in NCBI's Gene Expression Omnibus [46] and are accessible through GEO Series accession number GSE217303 (https://www.ncbi.nlm.nih.gov/geo/query/acc.cgi?acc=GSE217303). The datasets supporting the conclusions of this article are included within the article and its additional files.
