# Supplementary material for: Genome analysis of Cephalotrichum gorgonifer and identification of the biosynthetic pathway for rasfonin, an inhibitor of KRAS dependent cancer
Source: Fungal Biol Biotechnol. 2023 Jun 24;10:13. doi: 10.1186/s40694-023-00158-x (PMC10290801; doi:10.1186/s40694-023-00158-x)
Supplement: Supplementary file 1 — Additional file 1. Table S1: Information about oligonucleotides, strain verification, LC-HRMS analysis of KO strains, plasmid information, strain information, UV spectrum of rasfonin, nmr data of rasfoninm, Growth of C. gorgonifer at different temperatures. [file 40694_2023_158_MOESM1_ESM.pdf]

Additional Material 1:

Genome analysis of *Cephalotrichum gorgonifer* and identification of the biosynthetic pathway for rasfonin, an inhibitor of KRAS dependent cancer.

Andreas Schüller[1], Lena Studt-Reinhold[1], Harald Berger[1], Lucia Silvestrini[1§], Roman Labuda[2,3], Ulrich Güldener[4§§], Markus Gorfer[5], Markus Bacher[2,6], Maria Doppler[7,8], Erika Gasparotto[1,2§§§,] Arianna Gattesco[1,2], Michael Sulyok[7] and Joseph Strauss* [1,2]

[1] Institute of Microbial Genetics, Department of Applied Genetics and Cell Biology, University of Natural Resources and Life Sciences, Vienna (BOKU); Konrad Lorenz Strasse 24, 3430 Tulln a.d. Donau, Austria

[2] Research Platform Bioactive Microbial Metabolites (BiMM), Konrad Lorenz Strasse 24, 3430 Tulln a.d. Donau, Austria

[3] Department for Farm Animals and Veterinary Public Health, Institute of Food Safety, Food Technology and Veterinary Public Health; Unit of Food Microbiology, University of Veterinary Medicine Vienna, Veterinaerplatz 1, 1210 Vienna, Austria

[4] Department of Bioinformatics, Technical University of Munich, TUM School of Life Sciences Weihenstephan, Freising, Germany

[5] AIT Austrian Institute of Technology GmbH, Bioresources, 3430 Tulln, Austria

[6] Department of Chemistry, Institute of Chemistry of Renewable Resources, University of Natural Resources and Life Sciences Vienna (BOKU), Konrad-LorenzStraße 24, 3430 Tulln, Austria

[7] Department of Agrobiotechnology (IFA-Tulln), Institute of Bioanalytics and Agro-Metabolomics, University of Natural Resources and Life Sciences, Vienna (BOKU), Konrad Lorenz Strasse 20, 3430 Tulln a.d. Donau, Austria

[8]Core Facility Bioactive Molecules: Screening and Analysis, University of Natural Resources and Life Sciences, Vienna, 3430, Tulln a.d. Donau, Austria

§ present address: DGforLife, Operations – Research & Development, via Albert Einstein, Marcallo c.C., 20010, Milano, Italy

§§ present address: German Heart Center Munich, Technical University Munich, Lazarettstraße 36, 80636 Munich, Germany

§§§ present address: Faculty of Chemistry, Department of Biological Chemistry, University of Vienna, 1090 Vienna, Josef-Holaubek-Platz 2 Austria

*Corresponding Author: Joseph.strauss@boku.ac.at

Address: Joseph Strauss, Institute of Microbial Genetics, Department of Applied Genetics and Cell Biology, BOKU-University of Natural Resources and Life Sciences Vienna, BOKU-Campus Tulln, Konrad Lorenz Strasse 24, A-3430 Tulln/Donau, Austria. Tel. +43-147654-94420

1. Primer pairs used during this study

Primer Pair	Primer sequences (5'-->3'): Forward<br>Reverse	Info / Purpose
Primer pairs for yeast recombinational cloning or HiFi or for HDR transformation		
Q5_At_02774_1	GGCGAGGGCAACCAGCCCGGTGAGCGTTAGTTTTGCTGGCCGCATC	Template: pCBRS<br>Target: pAt-DNG02774
	ACCAAAACCACCCCAGTACATTAAAAACG	
Q5_At_02774_2	AGATTGTCGTTTCCCGCCTTCAG	Template: pCBRS<br>Target: pAt-DNG02774
	TATTTGAGAAGATGCGGCCAGCAAAACTAACGCTCACCGGGCTGGTTG	
Q5_At_02774_3	AGTTTAAACTGAAGGCGGGAAACGACAATCTGACTCTCGAAGCGATGT CAGCTC	Template: *C. gorgonifer* gDNA<br>Target: pAt-DNG02774
	TCAATATCAGTTAACGTCGACGGTATCGCGCGTCTCTAGCCCAGCC	
Q5_At_02774_4	CGATACCGTCGACGTTAACTGATATTGAAG	Template: pCBRS<br>Target: pAt-DNG02774
	GTTAACGTTAACTGGTTCCCGGTCG	
Q5_At_02774_5	ACCGGGAACCAGTTAACGTTAACattctttcgggcctgtatgttcg	Template: *C. gorgonifer* gDNA<br>Target: pAt-DNG02774
	TAACACATTGCGGACGTTTTTAAAGTACTGaattatgggagatagacggctctctgtc	
Q5-Cas9-scaff-1	acgccagggttttcccagtcacgacgGACGGCCAGTGCCAAGCTTAACG	Template: pFC331<br>Target: pCas9-scaff<br>Amplicon: 1/2 of AMA1 region
	CCACTGGACTCTCACCGTTTCCATTGG	
Q5-Cas9-scaff-2	TGGAAACGGTGAGAGTCCAGTGGGAAGATCTCATGGTCATAGCTGTTT CCGCTG	Template: pFC331<br>Target: pCas9-scaff<br>Amplicon: Cas9 gene
	ACTTAGAAATCACTCCATCCCGAGCTTAGCTTCAGACCTTGCGCTTCTT CTTGGGAGG	
Q5-Cas9-scaff-3	AGCTAAGCTCGGGATGGAGT	Template: A. fumigatus gDNA<br>Target: pCas9-scaff<br>Amplicon: T.gliN and gliA
	GTATCTGGTCGAAACATGTCTGCT	
Q5-Cas9-scaff-4	TCGACAAAAAGCAGACATGTTTCGACCAGATACCACGTGATTTAAATCG GTGATGTCTGCTCAAGCGGGGTAG	Template: A. nidulans gDNA<br>Target: pCas9-scaff<br>Amplicon: P.gpdA + R-sites
	ACGGAAGGACTTTGTCCAGTTGCATCGCACTAAGGCGTAAGCTCCCTA ATTGGCCCATCC	
Q5-Cas9-scaff-5	CTTAGTGCGATGCAACTGGACAAAGTCCTTCC	Template: psgRNA<br>Target: pCas9-scaff<br>Amplicon: P.gpdA+BsP1407I
	TTGCTATTTCTAGCTCTAAAACTGTACATGTGTAGATTCGTCTGGTACTG	
Q5-Cas9-scaff-6	AGTACCAGACGAATCTACACATGTACAGTTTTAGAGCTAGAAATAGC	Template: psgRNA<br>Target: pCas9-scaff<br>Amplicon: sgRNA -scaffold + T.tef
	accgcctctccccgcgcgttggccgattcattaaCATTGTTATCTTCAATGTCTGTCATA GCATTGCGTGAAGC	
Q5-Cas9-scaff-7	tcggccaacgcgcggggag	Template: pRS426<br>Target: pCas9-scaff<br>Amplicon: ampR and ColE1 ori
	tgactgggaaaaccctggcgtcaggtggcacttttcggggaaatg	
Q5-Cas9-hph	ACAAAAAGCAGACATGTTTCGACCAGATACCTATTCCTTTGCCCTCGGA CGAGTGCTG	Template: pCSN44<br>Target: pCas9-hph<br>Amplicon: hygromycin marker
	ACTAACAGCTACCCCGCTTGAGCAGACATCACCGATGAAAAAGCCTGA ACTCACCGCGACGTC	
HDR-Fragment	AGTTTAAACTGAAGGCGGGAAACGACAATCTGACTCTCGAAGCGATGT CAGCTC	Template: pAt-DNG02774<br>Amplicon: hph cassette with homology regions of DNG02774<br>35 cycles- 5 min elongation-60°C
	TAACACATTGCGGACGTTTTTAAAGTACTGaattatgggagatagacggctctctgtc	
Primer pairs for transformant verification		

At-HDR_Diag	gttaccggtatcatgcttgagagcct	Strains: Cg-02774-At-1, Cg-HDR-02774-1 Amplicon: Outside of homology regions (i.e. whole insert)
	TGATTTGGGTGTCAACGTCATACTGGC	
Cas9-Diag	TCAGCTCGTTGACACTGGAAGCAC	Strain: Cg-Cas9-02774-1 Amplicon: Promoter and 5'-end of DNG02774
	tggcctaactccgatcaagctacagc	
	TGGTTCAAGGCGCTCCCATCACG	

Table S 1 Primer paris used during this study

2. Diagnostic PCRs of Cg-02774-At-1 and Cg-02774-At-1

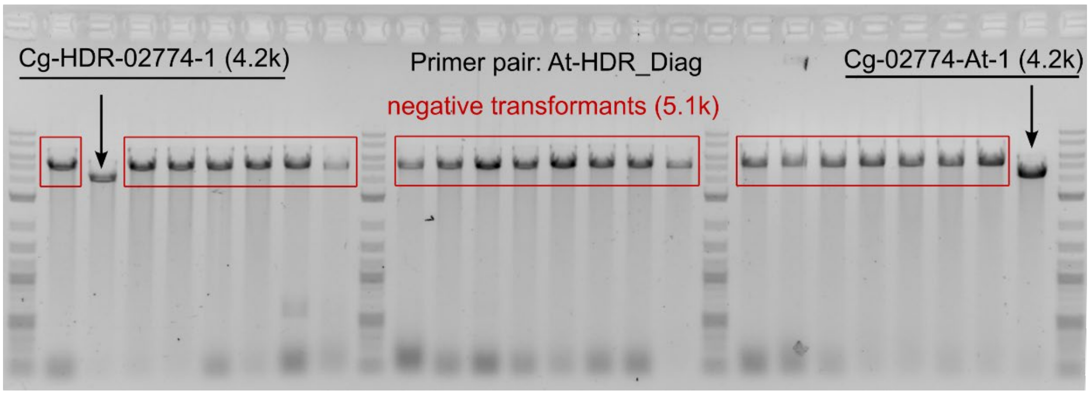

Figure S 1 Agarose gel electrophoresis of diagnostic PCRs of transformants from Agrobacterium tumefaciens and homology directed repair transformations. The primer pair At-HDR_Diag (Table S 1) was used which results in a PCR product of 4221 base-pairs in case of a successful transformation. Otherwise the PCR product is 5085 base-pairs long. Negative transformants are indicated with red box and text. Positive transformants are marked by arrows and the corresponding name. The ladder in use is the 1 kb Plus DNA Ladder from NEB (Art. No.: N3200S)

3. LC-HRMS Data of Knockout strain Cg-Cas9-02774-1 and wildtype strain NG_p51

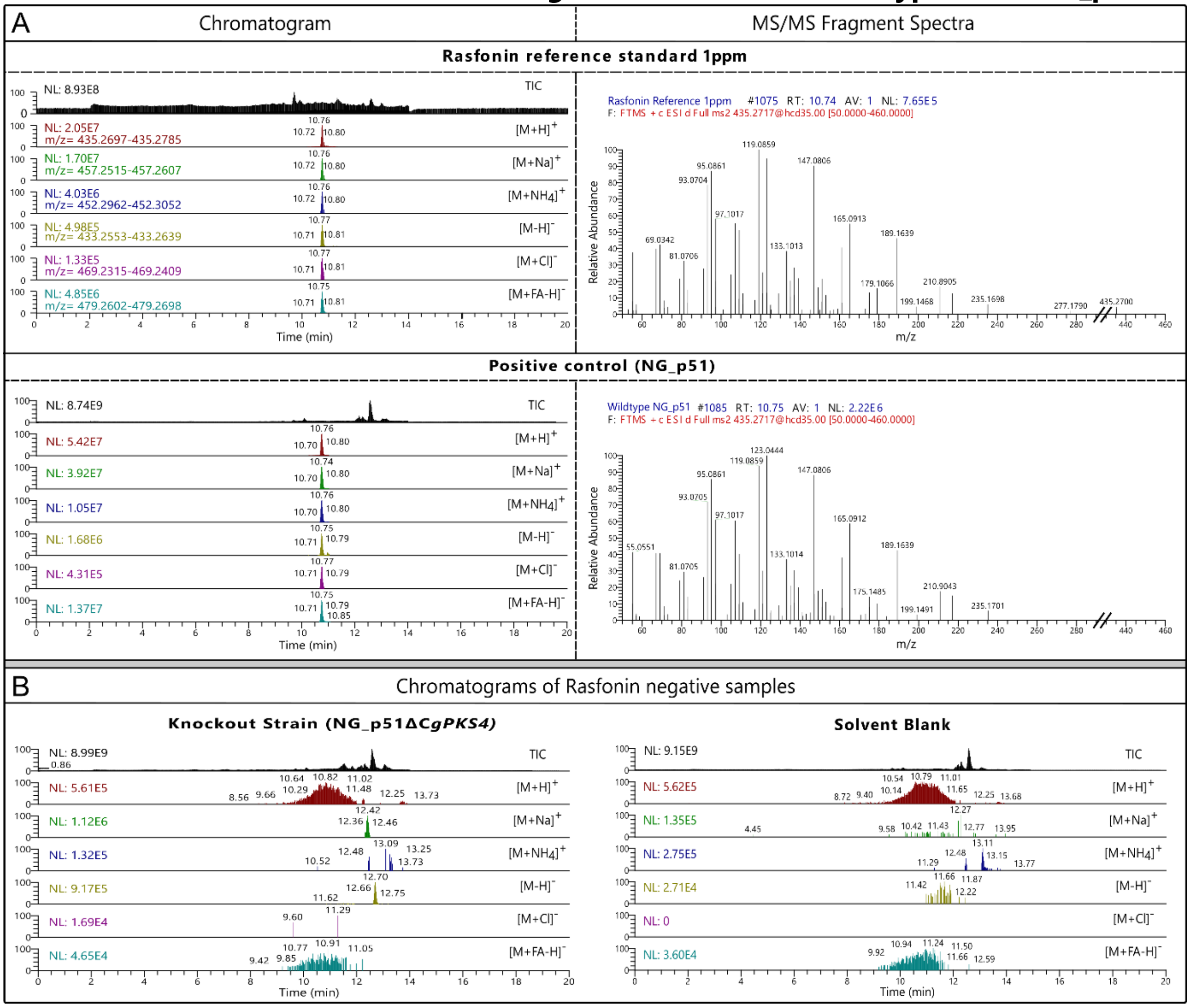

Figure S 2 Detection of rasfonin in wildtype strain Isolate NG_p51 and knockout strain NG_p51ΔCgPKS4 via LC-HRMS(/MS) and ionisation in fast polarity switching mode. ***A:*** *Comparison of chromatographic information (total ion chromatogram and extracted ion chromatograms of positive ([M+H]+, [M+Na]+, [M+NH4]+) and negative [M-H]-, [M+Cl]-, [M+FA-H]-) ions; left panel) and MS/MS fragment spectra (right panel) of the 1ppm rasfonin standard (top) and the wildtype NG_p51 media extract (Positive control; bottom). All spectra show the specific pattern for rasfonin which confirms that rasfonin is produced by the wildtype strain.* ***B:*** *Chromatographic information of the knockout strain (NG_p51ΔCgPKS4) and the solvent blank. Both chromatograms confirm that rasfonin is absent in the media extract of the knockout strain. Both chromatograms show similar signals which indicates that they originate from the extraction solvent (i.e.: ACN). The experiment confirms that CgPKS4 of C. gorgonifer (gene DNG_02774) is involved in the production of Rasfonin.*

4. Plasmids that have been constructed during this study

Plasmid	Main Elements	Backbone
pAt-DNG02774	replacement cassette DNG02774 - *A. tumefaciens* transformation backbone	pCBRS
pCas9-DNG02774-sgRNA5	pCas9-hph plus sgRNA for DNG02774	pCas9-hph
pCas9-hph	pCas9-scaff plus hygromycin marker	pCas9-scaff
pCas9-scaff	Cas9 - 1/2 AMA1 - sgRNA scaffold	-

Table S 2 Plasmids constructed and used during this study. All plasmids can be retrieved from the additional files in genbank format.

5. All fungal strains used during this study

Strain	Genotype	Parent strain	Transformed vector
C. gorgonifer NG_p51	wildtype strain	-	-
Cg-Cas9-02774-1	ΔDNG_02774	C. gorgonifer NG_p51	pCas9-DNG02774-sgRNA5
Cg-At-02774-1	DNG_02774::hph	C. gorgonifer NG_p51	pAt-DNG02774
Cg-HDR-02774-1	DNG_02774::hph	C. gorgonifer NG_p51	PCR (HDR-Fragment)

Table S 3 Fungal strains used during this study. All strains apart from the wildtype NG_p51 have been generated during this study

6. UV absorption spectrum of purified rasfonin

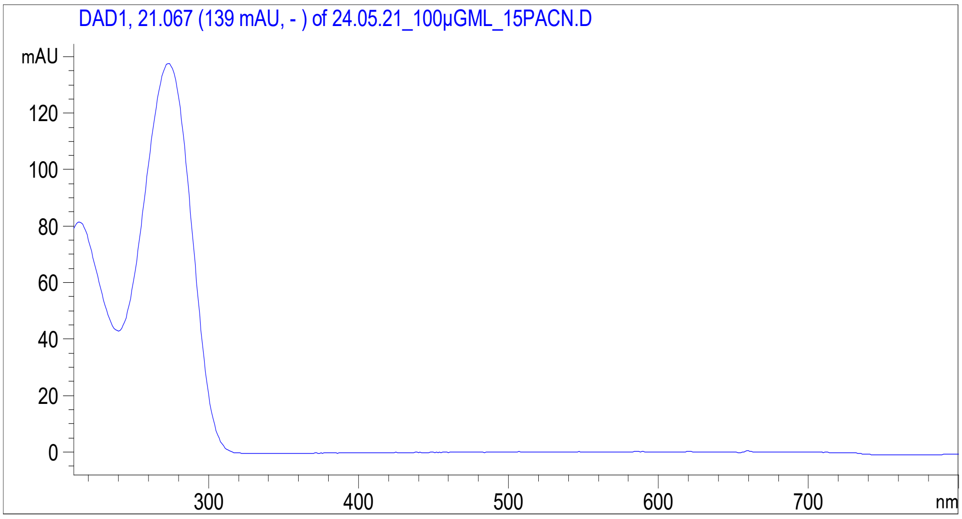

Figure S 3 UV- spectrum of purified rasfonin. Max absorption was measured at 280 nm which was subsequently used for detection during HPLC analysis.

7. ^{1}H and ^{13}C nmr data of rasfonin in CDCl3

	^{1}H	^{13}C
1	-	163.29
2	6.21, d, 1H, *J* = 9.6	124.93
3	7.04, dd, 1H, *J* = 9.6; 6.0	140.58
4	5.34, dd, 1H, *J* = 6.0; 2.5	61.70
5	4.13, dd, 1H, *J* = 8.9; 2.5	83.28
6	2.18, m, 1H	31.37
7	1.21, ddd, 1H, *J* = 13.4; 9.0; 3.9	39.94
	1.03, ddd, 1H, *J* = 13.4; 10.0; 4.8	
8	1.68, m, 1H	27.85
9	2.06, br.d, 1H, *J* = 13.2	46.28
	1.44, dd, 1H, *J* = 13.2; 9.8	
10	-	134.18
11	5.12, br.q, 1H, *J* = 7.1	120.04
12	1.54, br.d, 3H, *J* = 7.1	13.33
13	1.15, d, 3H, *J* = 6.6	18.85
14	0.78, d, 3H, *J* = 6.5	20.59
15	1.52, br.s, 3H	15.53
1’	-	168.08
2’	5.81, d, 1H, *J* = 15.6	115.01
3’	7.34, dd, 1H, *J* = 15.6; 0.5	150.96
4’	-	134.63
5’	5.77, br.d, 1H, *J* = 9.9	143.28
6’	2.88, m, 1H	39.22
7’	1.79 + 1.61, each m, 1H	34.74
8’	3.73, dt, 1H, *J* = 10.6; 5.5	60.60
	3.60, m, 1H	
9’	1.83, d, 3H, *J* = 1.2	12.59
10’	3.60, m, 2H	65.81

Table S 4: ^{1}H and ^{13}C nmr data of rasfonin in CDCl3

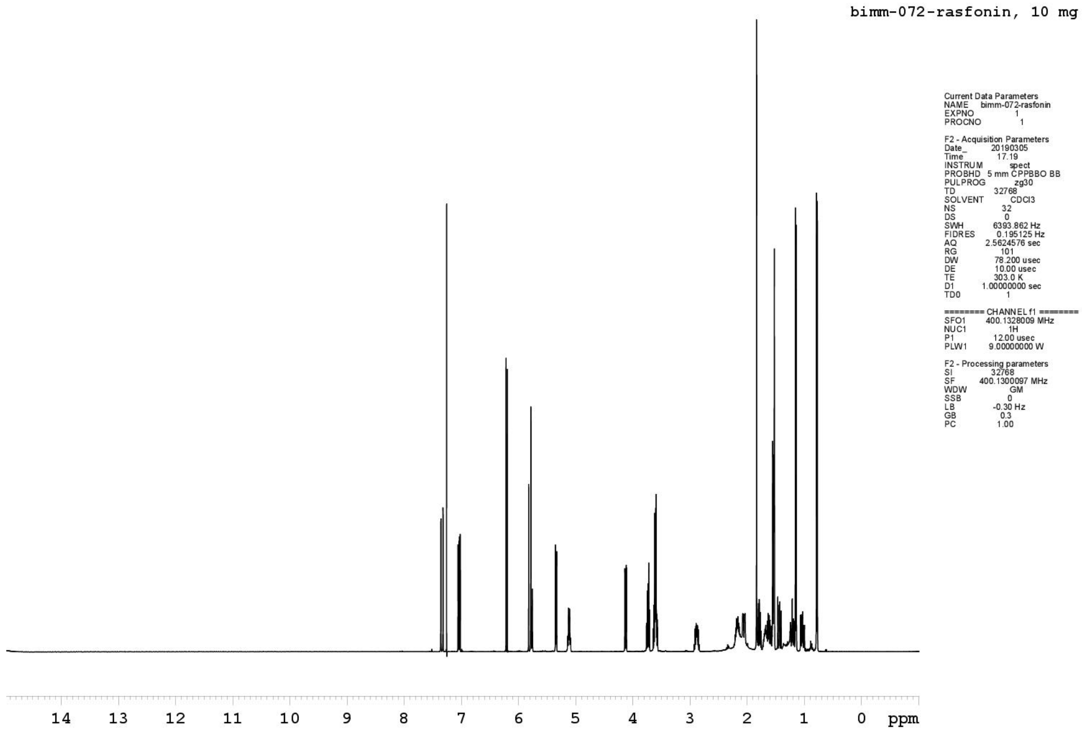

Figure S 4 ^{1}H NMR spectrum of rasfonin

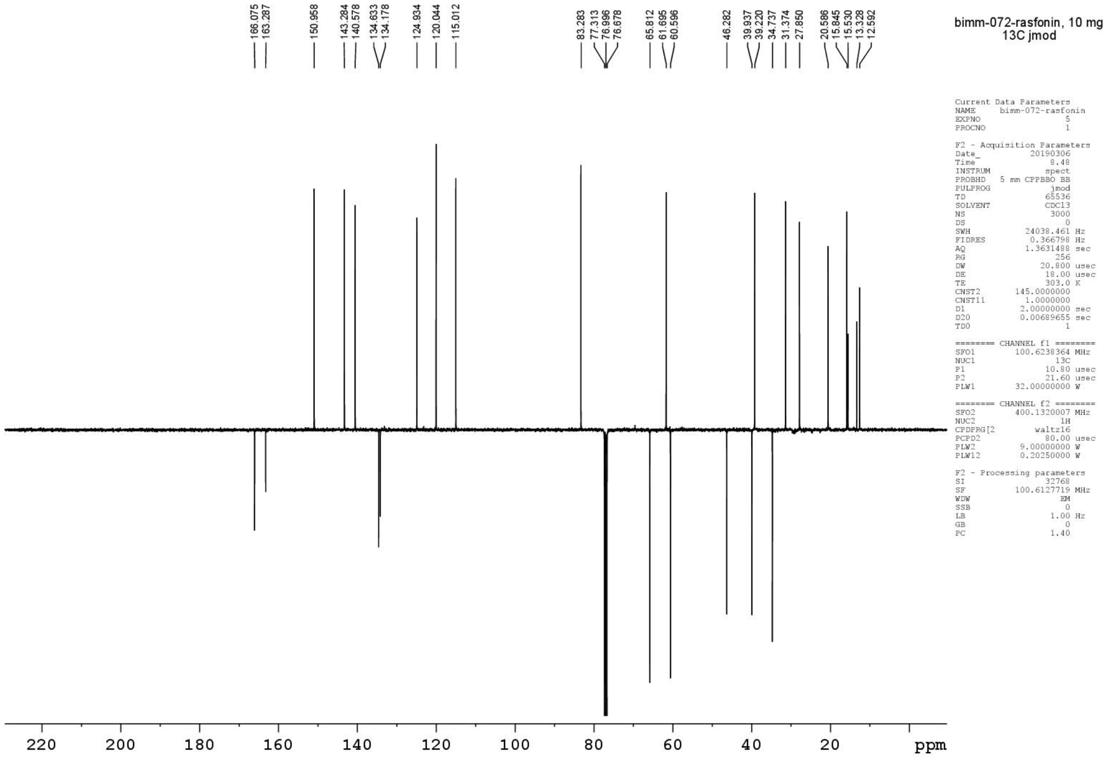

Figure S 5 ^{13}C NMR spectrum of rasfonin

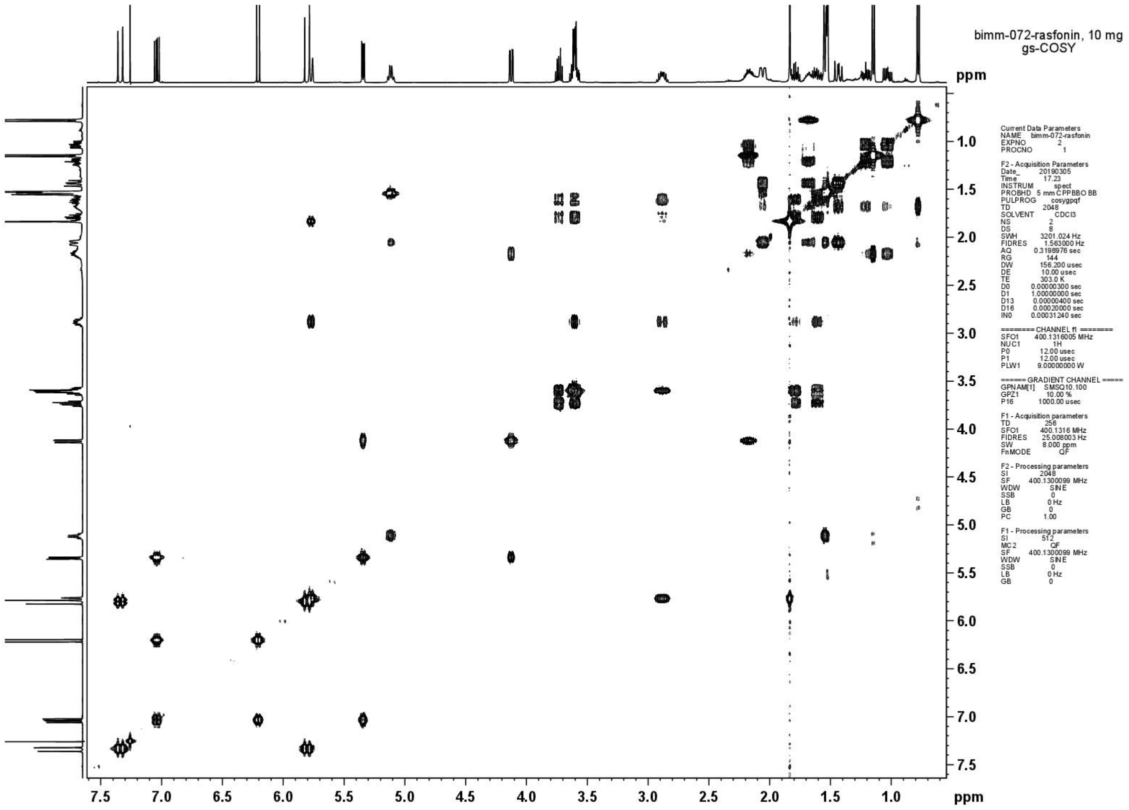

Figure S 6 COSY spectrum of rasfonin

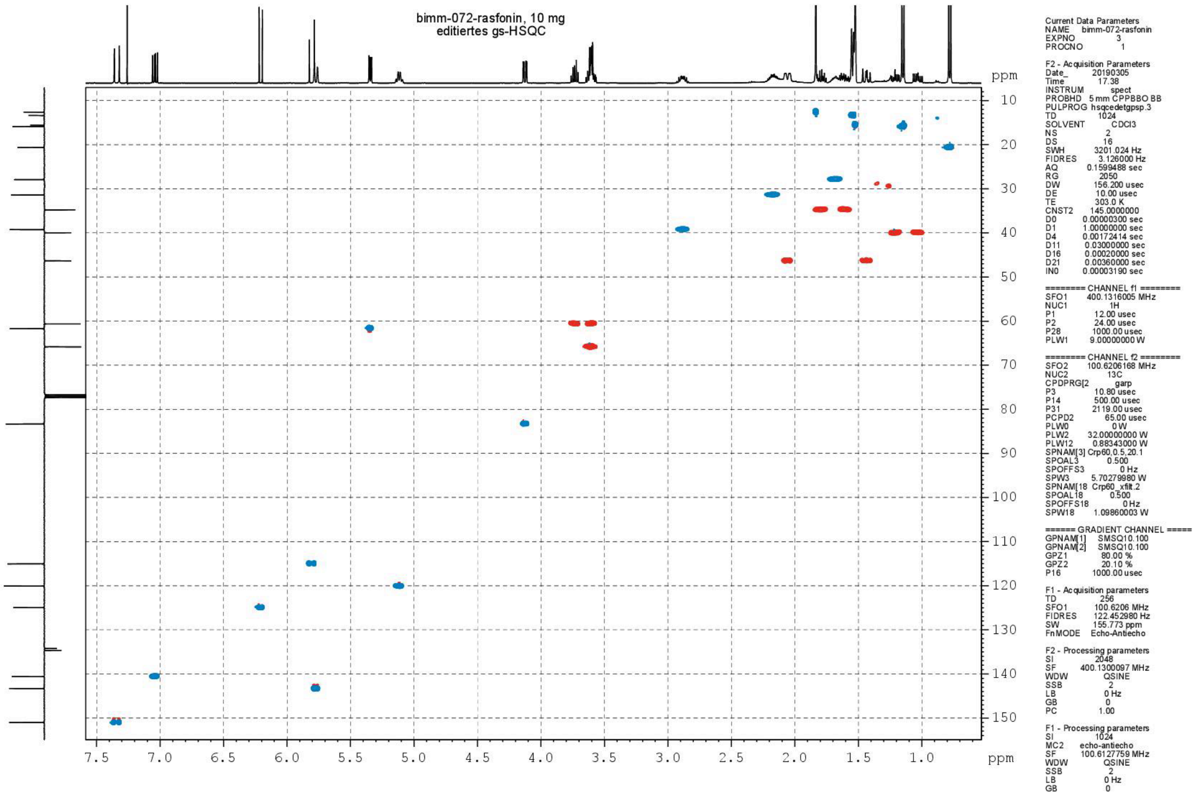

Figure S 7 HSQC spectrum of rasfonin

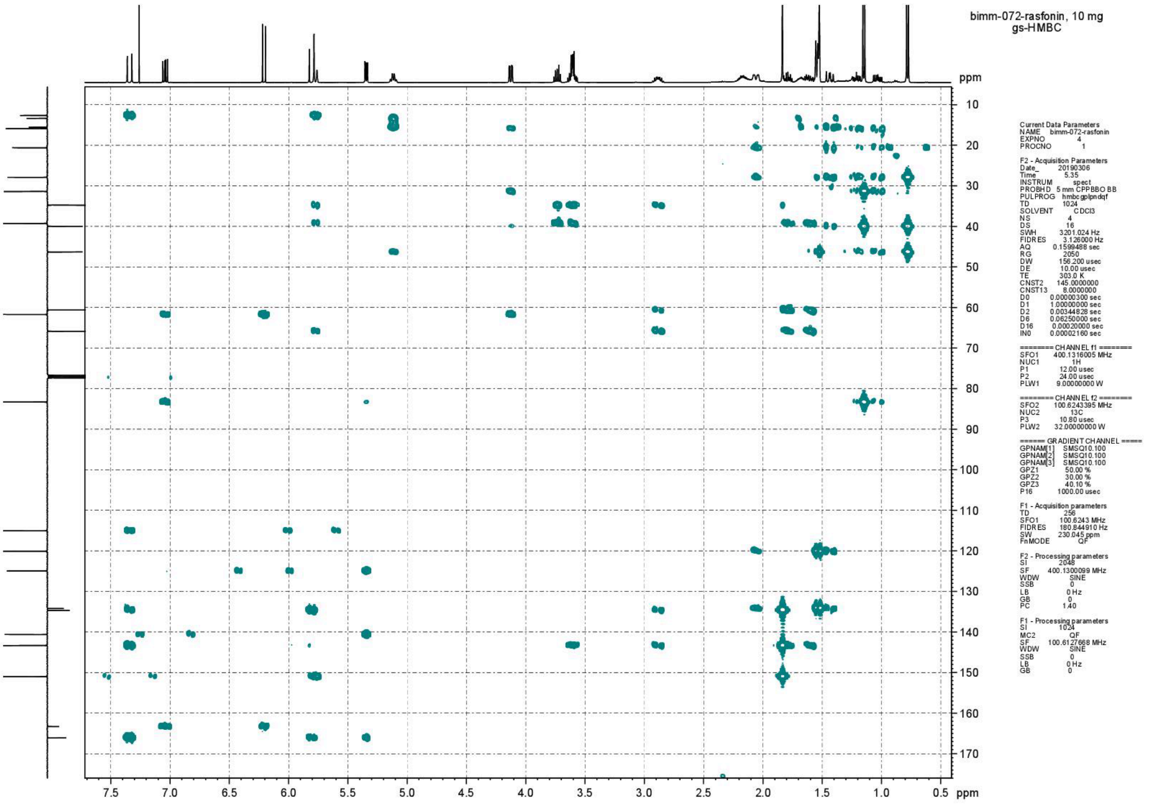

Figure S 8 HMBC spectrum of rasfonin

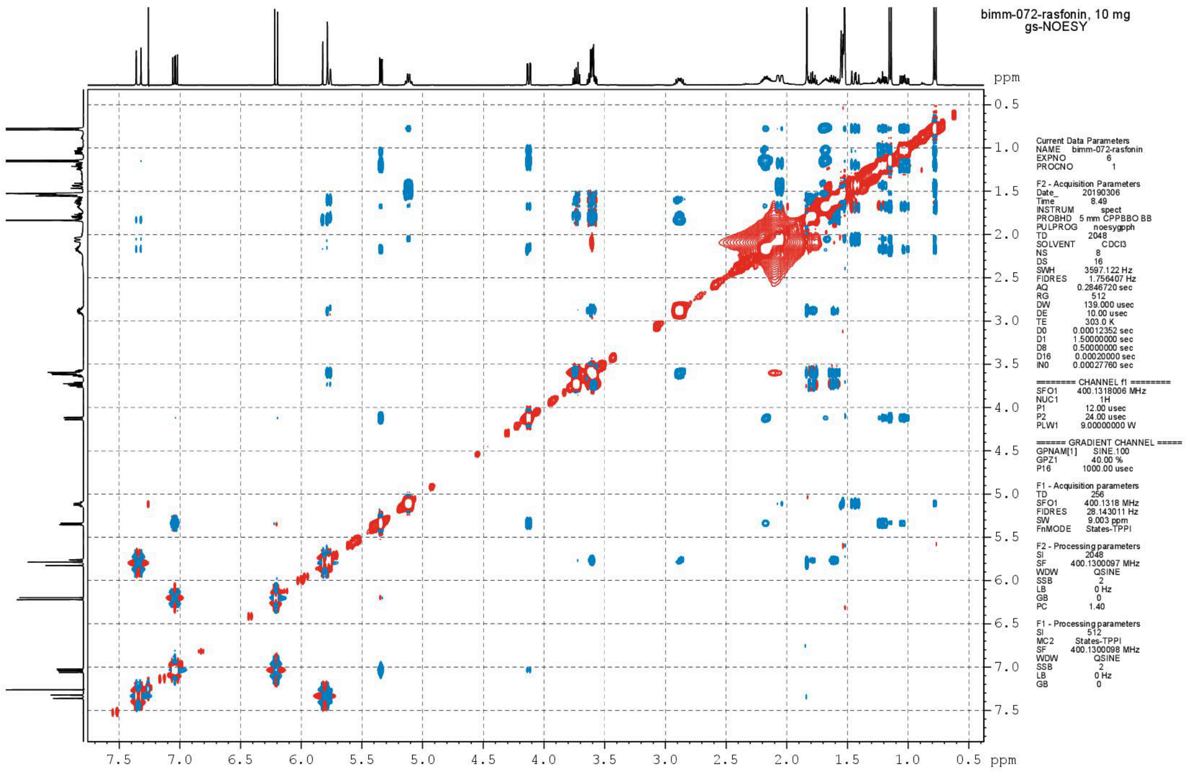

Figure S 9 NOESY spectrum of rasfonin

8. Growth of *C. gorgonifer* at different temperatures

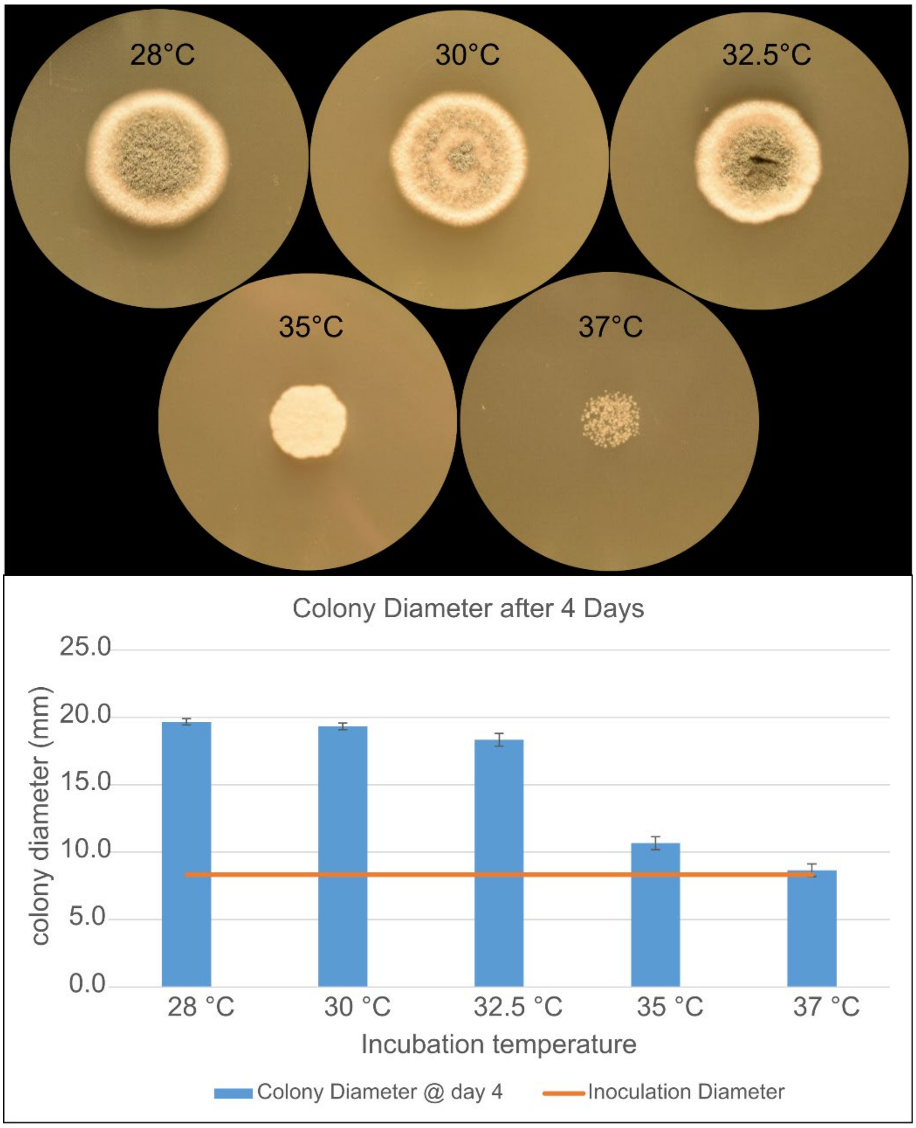

Figure S 10 Radial growth of C. gorgonifer NG_p51 on MEA petri dishes at different temperatures after 4 days. Optimal growth was at 28°C. At 35°C, radial growth was strongly impaired while at 37°C radial growth was inhibited. However, within the inoculation spot, spores at 37°C were germinating and mycelia density was increasing over the 4 days of incubation
